# Thiamethoxam at environmentally relevant concentrations induces neurotoxicity in zebrafish larvae through binding with multiple receptors

**DOI:** 10.1016/j.eehl.2024.12.002

**Published:** 2025-01-17

**Authors:** Xiuwen Li, Hanbing Zhao, Minjuan Gong, Feng Zhang, Shengnan Liu, Zepeng Zhang, Yide He, Henner Hollert, Xiaowei Zhang, Wei Shi, Qing Zhou, Aimin Li, Peng Shi

**Affiliations:** a State Key Laboratory of Pollution Control and Resource Reuse, School of Environment, Nanjing University, Nanjing 210023, China; b Key Laboratory of Mesoscopic Chemistry of Ministry of Education (MOE), School of Chemistry and Chemical Engineering, Nanjing University, Nanjing 210023, China; c School of Environmental Science and Engineering, Nanjing Tech University, Nanjing 211816, China; d Department Evolutionary Ecology & Environmental Toxicology, Goethe University, Frankfurt 60438, Germany; e Department Environmental Media Related Ecotoxicology, Fraunhofer Institute for Molecular Biology and Applied Ecology IME, Schmallenberg 57392, Germany

**Keywords:** Thiamethoxam, Zebrafish, neurotoxicity, Multi-omics, Multi-receptors

## Abstract

Thiamethoxam (THM) is one of the most widely used insecticides globally, which was designed to selectively target nicotinic acetylcholine receptors (nAChRs) in the insect nervous system and is generally considered safe for non-targeted organisms. However, increasing evidence has demonstrated its neurotoxicity in aquatic organisms, though the underlying mechanisms, especially at environmentally relevant concentrations, remain largely unclear. In this study, the swimming distance of zebrafish was significantly shortened by 14.06%–21.64% after exposure to THM at 10–1000 ng/L. This behavioral impairment may result from the damage to nervous and visual systems, as confirmed by notable apoptosis, histological analysis of the eyes, and differential expression of numerous genes. Molecular docking and biomarkers assays found that THM can bind with nAChR and multiple hormone receptors, with binding energies varying from −3.75 to −6.74 kcal/mol. Consequently, the concentrations of a neurotransmitter (acetylcholine) and related hormones (cortisol, triiodothyronine, thyroxine, and thyroid-stimulating hormone) were significantly affected. Further investigations using a weighted gene correlation network and metabolomics suggest that THM may enter the cell via endocytosis and bind with multiple hormone receptors, potentially activating the MAPK signaling pathway. This activation may disrupt purine and pyrimidine metabolism in the cell nucleus, ultimately leading to cell apoptosis and neurotoxicity. This study reveals that THM, even at environmentally relevant concentrations, poses neurological risks to zebrafish and underscore the need for urgent attention to the ecological impacts of THM in aquatic environments.

## Graphical abstract

**Figure:**
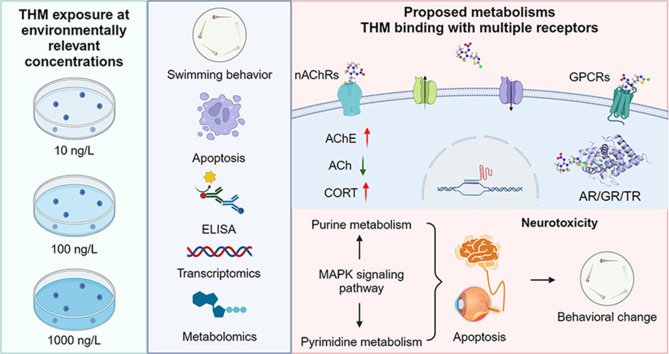

## 1 Introduction

Thiamethoxam (THM) is a second-generation neonicotinoid insecticide developed to control a wide range of sucking and chewing pests [1]. Renowned for its broad spectrum of insecticidal activity, versatility in usage, and high efficiency, THM has become one of the most popular insecticides since its introduction in 1998 [2]. However, concerns over its ecotoxicity led the European Commission to restrict its use for seed treatment in 2013 [3], followed by a complete ban on outdoor use in 2018 [4]. Despite these restrictions, many countries, including China, have no similar regulations yet.

The widespread use of THM, coupled with its high water-solubility, has inevitably led to its mounting release into the environment, particularly through agricultural runoff [5]. THM has half-lives (DT_50_) of 7–72 days in soil and 11.5 days in water, demonstrating resistance to hydrolysis under neutral or acidic pH conditions and in anaerobic environments [6]. Consequently, THM has been frequently detected in surface waters worldwide. For instance, THM concentrations reached up to 1.34 μg/L in Canada and 118 ng/L in Portugal, respectively [1,7]. Our previous research identified THM as the most prevalent neonicotinoid insecticide in the lower reaches of the Yangtze River, China, with concentrations ranging from 0.29 to 48.15 ng/L [8].

The frequent occurrence of THM in aquatic systems raises concerns about its ecotoxicity. Although THM was designed to selectively bind to nicotinic acetylcholine receptors (nAChRs) of insects, while being relatively mild on vertebrate nAChRs [9], an increasing number of studies have reported adverse effects of THM on aquatic organisms. For instance, parental exposure to THM at 50 and 500 ng/L for 144 days, not only affected the reproductive system of F0 zebrafish, but also altered sex hormones levels and developmental processes in F1 zebrafish [10]. Neurotoxicity is a major concern when considering its toxic mechanism. THM altered locomotion in zebrafish larvae at a relatively high concentration of 0.1 mg/L [11]. Chronic exposure to THM at 155 μg/L resulted in significant impairment of predator escape response, while exposure to THM at 0.02–14.61 μg/L negatively affected the foraging efficiency of fathead minnows (*Pimephales promelas*) [12]. More importantly, environmentally relevant concentrations of THM (1.5 μg/L) can induce neurotoxicity in zebrafish during early developmental stages [13]. However, the exact mechanisms behind this neurotoxicity remain unclear, warranting further explorations.

A previous study reveals that THM has a low affinity for nAChR in zebrafish, but still induces neurobehavioral changes [14], suggesting that other toxic mechanisms may be involved beyond nAChR binding. Oxidative damage is recognized as a common pathway for chemical-induced neurotoxicity [15], and THM has been reported to significantly increase levels of reactive oxygen species (ROS), and superoxide dismutase (SOD) in zebrafish liver at 0.3 mg/L [16]. However, whether environmentally relevant concentrations of THM can trigger oxidative damage remains to be verified. Additionally, hormone receptors such as adrenoceptors (ARs), glucocorticoid receptors (GRs), and thyroid receptors (TRs) are also implicated in pollutant-induced neurotoxicity, especially at low concentrations [[17], [18], [19], [20]]. One study found that chronic exposure to THM impaired body development and caused hepatic tissue necrosis in adult Chinese rare minnow (*Gobiocypris rarus*), possibly through the binding of estrogen receptor α (ERα), AR and TRα via hydrogen bonding [21]. Notably, THM at environmentally relevant concentrations altered the expression of genes involved in behavior, hormone synthesis and neurodevelopment in zebrafish larvae [22]. This suggests that hormone receptors may play a crucial role in mediating neurotoxicity caused by THM at the molecular level. In summary, THM at environmentally relevant concentrations may induce neurotoxicity in zebrafish through multiple mechanisms, and a comprehensive investigation is needed to fully elucidate the complex mechanisms.

Here, we exposed zebrafish to THM at environmentally relevant concentrations (10–1000 ng/L) and employed a combination of behavioral assessments, histological analysis, biomarker measurements, molecular docking, and multi-omics analyses. The goal of this research is to deepen our understanding of THM's effects on aquatic ecosystems, and to emphasize the urgent need for evaluating its ecological risks.

## 2 Materials and methods

### 2.1 Chemicals

THM (C_8_H_10_ClN_5_O_3_S) was obtained from Energy Chemical. CaCl_2_·2H_2_O, MgSO_4_·7H_2_O, NaHCO_3_, and KCl were supplied by Shanghai Lingfeng Chemical Reagent Co., Ltd. Tricaine was provided by TCI Shanghai. The purities of these chemicals all exceed 98%.

### 2.2 Zebrafish embryo exposure

Wild-type zebrafish (Tübingen line) embryos were purchased from Nanjing EzeRinka Biotechnology Co., Ltd. (Nanjing, China). THM stock solutions were prepared to achieve nominal concentrations of 10, 100, and 1000 ng/L by dissolving THM into the embryo culture medium, which consists of 0.294 g/L CaCl_2_·2H_2_O, 0.1233 g/L MgSO_4_·7H_2_O, 0.063 g/L NaHCO_3_, and 0.0055 g/L KCl. Exposures were conducted using glass Petri dishes (200 mm × 28 mm) filled with 500 mL of exposure solution, housing 300 embryos per dish for enzyme-linked immunosorbent assay (ELISA) and multi-omics analysis. For histological analysis, locomotion and apoptosis assessments, 36 embryos per THM concentration were placed in individual wells of a 96-well plate, with each well containing one embryo and 200 μL of exposure solution. The embryos were incubated at 28 ± 0.5 °C with a 14/10-h light–dark cycle. Embryo culture medium served as the control group. For each analysis (apoptosis, histology, ELISA, and multi-omics), three replicates were used, while locomotion assessments were performed with 36 replicates. Embryos were exposed from 6 h post fertilization (hpf, gastrula stage) to 120 hpf (larva stage), a non-protected developmental period as per EU Directive 2010/63/EU [23]. During the half-static exposure, two thirds of the exposure solution was renewed every 24 h in each Petri dish or well, including the control group, following OECD 210 guidelines [24]. Before each renewal, 50 mL of exposure solutions were collected for THM concentration analysis, and the pretreatment and analytical methods were detailed in Text S1. The actual THM concentrations detected in solutions with nominal concentrations of 10, 100 and 1000 ng/L were 9.5 ± 1.0, 87.3 ± 4.9, and 900.6 ± 48.9 ng/L, respectively (Table S1). THM was below the detection limit in the control group. Thus, nominal concentrations were used in the subsequent analysis.

### 2.3 Locomotion, apoptosis, and histological analysis

At 120 hpf, swimming behavior was observed and recorded using the Zebralab Video-Track system (ViewPoint Life Science, France). The system was equipped with a PointGray IEEE-1394 camera (model GRAS-03K2M−C, 30 fps), integrated LED lights, and an infrared filter. As described by Noyes et al. [25], the swimming distance per minute was monitored and recorded in response to a 5-min period of continuous light, followed by a 10-min dark period stimulation test, with a 5-min interval, and concluding with an additional 5 min of continuous light. After completing the locomotion analysis, three larvae from each concentration group were randomly selected and euthanized in 0.016% tricaine on ice. The TUNEL assay for whole organism analysis was conducted using the TMR-RED *in situ* cell death detection kit (Roche, Basel, Switzerland) following the manufacturer's protocol. Apoptosis was then visualized and recorded using a laser scanning microscope (Nikon 120c, Japan). Three larvae from each concentration group were randomly selected for histological analysis. After washing with PBS three times, the larvae were fixed in 4% paraformaldehyde at 4 °C for 24 h. The zebrafish larvae were embedded in paraffin after dehydration using sucrose. Tissue sections, 3–5 μm in thickness were sliced and stained with hematoxylin and eosin. Imaging was performed using a biological microscope (Nikon 120c, Japan).

### 2.4 Enzyme-linked immunosorbent assay (ELISA)

At 120 hpf, zebrafish larvae were euthanized on ice and washed three times with phosphate buffer saline (PBS, 0.1 M, pH 7.4, KeyGEN, China). Homogenization was performed in PBS using a high throughput tissue crusher (SCIENTZ-48, China) at 50 Hz for 6 min, followed by centrifugation at 6000 rpm for 10 min at 4 °C. The supernatants were collected and stored at −20 °C until assayed. Biomarkers including acetylcholinesterase (AChE), acetylcholine (ACh), ROS, SOD, catalase (CAT), adrenaline (ADR), cortisol (CORT), triiodothyronine (T3), thyroxine (T4), and thyroid-stimulating hormone (TSH) were quantified according to the protocols provided with the ELISA kits (ZCIBIO, China).

### 2.5 Molecular docking

Molecular docking analyses were performed to assess the binding affinities of THM with the key target receptors, including nAChR, G-protein-coupled receptor (GPCR), ARα, ARβ, GR, TRα, and TRβ. The 3D structures of these receptors in zebrafish were generated through homology modeling strategy using the SWISS-MODEL (https://swissmodel.expasy.org/), while the 3D structure of THM (CID number: 5821911) was obtained from PubChem (https://pubchem.ncbi.nlm.nih.gov/). Binding energy calculations were conducted using AutoDock Tools (version 1.5.6), and the molecular interactions between THM and receptor amino acids were visualized with PyMOL (version 1.3) based on the lowest docking energy [17]. Further details on the homology modeling and docking procedures are available in Text S2, Table S2 and Figs. S1–S7.

### 2.6 RNA isolation, sequencing and transcriptomic analysis

At 120 hpf, 100 larvae from each dish were rinsed three times with PBS and rapidly frozen in liquid nitrogen. Total RNA was extracted using TRIzol reagent (Invitrogen, USA) and further purified with the Qiagen RNeasy miniRNA cleanup kit (Qiagen, USA) following the manufacturer's guidelines. RNA quality was evaluated through the 2100 Bioanalyzer (Agilent) and quantified with the ND-2000 (NanoDrop Technologies). Only RNA samples that met rigorous quality control criteria (OD260/280 = 1.8–2.2, OD260/230 ≥ 2.0, RNA Integrity Number (RIN) ≥ 6.5, 28S:18S ≥ 1.0, and yield 1 μg) were selected for sequencing library construction. For each experimental condition, three RNA replicates were processed by Genepioneer Biotechnologies Inc. (Nanjing, China) for RNA-Seq using the Illumina TruSeqTM RNA sample preparation Kit (San Diego, CA) with 1 μg of total RNA. After library quantification using the TBS380 fluorometer, paired-end RNA-Seq libraries were sequenced on the Illumina HiSeq X Ten or NovaSeq 6000 platform, generating reads with a length of 2 × 150 bp.

The raw sequencing data was processed using Trimmomatic software (version 0.36) with default settings to remove noise and ensure data quality [26]. Clean reads were subsequently aligned to the zebrafish reference genome (GRCz11) using HISAT2 (version 2.0.4). After quality control using Trimmomatic (version 0.38), high-quality sequencing data were analyzed to identify differentially expressed genes (DEGs) through the edgeR package (version 4.0) in R software (version 4.2.1). Principal coordinate analysis (PCoA) was performed using the same version of R. DEGs were defined as those with a fold change (FC) greater than ± 2.0 and a *p*-value below 0.05 [26]. The biological relevance of the DEGs was explored utilizing the Gene Ontology (GO) and Kyoto Encyclopedia of Genes and Genomes (KEGG) pathway analyses, facilitated by the DAVID Bioinformatics Resources 6.8 platform (https://david.ncifcrf.gov/). Weighted Gene Co-Expression Network Analysis (WGCNA) was conducted using the WGCNA package in R, and potential hub genes within each module were subjected to KEGG pathway analysis, with thresholds set at gene significance >0.2 and module membership >0.8 [27]. Modules were identified through the dynamic tree-cut algorithm, and those showing statistical significance (*p* < 0.05) for each trait were selected for further analysis. Genes associated with neuronal and visual systems were curated based on previously published literature and data from the Zebrafish Information Network database (https://zfin.org/) (Table S3). All transcriptomic data were deposited in the National Center for Biotechnology Information (NCBI) Sequence Read Archive (PRJNA1073005).

### 2.7 Quantitative real-time polymerase chain reaction (qRT-PCR)

Five genes (*elavl3*, *adra2b*, *gap43*, *opn1sw1*, and *rho*) involved in nervous and visual systems were selected for qRT-PCR. Their corresponding primers (10 μM) are listed in Table S4. The cDNA was synthesized from the same RNA samples used for RNA-Seq analysis, using a high-capacity cDNA reverse transcription kit (Thermo Fisher Scientific, USA). qRT-PCR was performed following the manufacturer's protocol for the TransStart Top Green qPCR SuperMix (TransGen Biotech, China), utilizing 40 amplification cycles on a real-time PCR system (Thermo Fisher Scientific, USA). The thermal cycling conditions for qRT-PCR are provided in Table S5. Relative gene expression levels were determined using the 2^−ΔΔCT^ method, with *β-actin* serving as the reference gene, as its expression remained stable under THM exposure [28,29].

### 2.8 Non-targeted metabolomics analysis

At 120 hpf, 100 larvae were rinsed with PBS and rapidly frozen with liquid nitrogen. Subsequently, 400 μL of a methanol:water (4:1, v/v) solution with 20 mg/L L-2-chlorophenylalanin as an internal standard was added for metabolite extraction. The extraction was performed at −10 °C using a high throughput tissue homogenizer (SCIENTZ-48, China) set to 50 Hz for 6 min, followed by sonication at 40 kHz for 30 min at 5 °C. The samples were then stored at −20 °C for 30 min to allow protein precipitation. Afterwards, centrifugation was carried out at 12,000 rpm for 15 min at 4 °C.The supernatant was carefully collected for analysis using ultra-high performance liquid chromatography-tandem time-of-flight mass spectrometry (UPLC-TripleTOF, AB SCIEX, USA). Specific details on the chromatographic and MS conditions are presented in Text S3 and Table S6.

The raw metabolic data were preprocessed using Progenesis QI software (Waters Corporation, Milford, USA), and metabolite identification was carried out through searches in several databases, including HMDB (http://www.hmdb.ca/), Metlin (https://metlin.scripps.edu/), and Majorbio Database. Significantly differentially changed metabolites (DCMs) were identified using variable importance in projection (VIP) scores from the orthogonal partial least squares discriminant analysis (OPLS-DA) model and the *p*-value from the Student's t-test. Metabolites with VIP score greater than 1 and *p*-value less than 0.05 were deemed as significantly altered. These DCMs were then mapped to their corresponding biochemical pathways through metabolic enrichment and pathway analysis using database (KEGG, http://www.genome.jp/kegg/). Additionally, integrated transcriptomic and metabolomic analyses were conducted through the joint pathway analysis module in MetaboAnalyst 5.0 (https://www.metaboanalyst.ca/MetaboAnalyst).

### 2.9 Statistical analysis

The fluorescence intensity of apoptosis was quantified using Image J online (https://ij.imjoy.io/) [30] and normalized by dividing the integrated density by the area. Apoptosis fluorescence results, along with biomarkers measurements, were reported as average (AVG) ± standard deviation (SD). Prior to statistical analysis, normal distribution and homogeneity of variance were tested through Shapiro–Wilk's test and Levene's tests, respectively. Parametric tests were performed for data with normal distribution and variance homogeneity. Otherwise, nonparametric tests were conducted. Statistical comparisons were conducted between the control and exposure groups, using a one-way analysis of variance (ANOVA) with Dunnett's post-test in GraphPad Prism 8.3, with significance set at *p* < 0.05.

## 3 Results

### 3.1 THM induced the photomotor response change, apoptosis, and histological change in zebrafish larvae

**Fig. 1:**
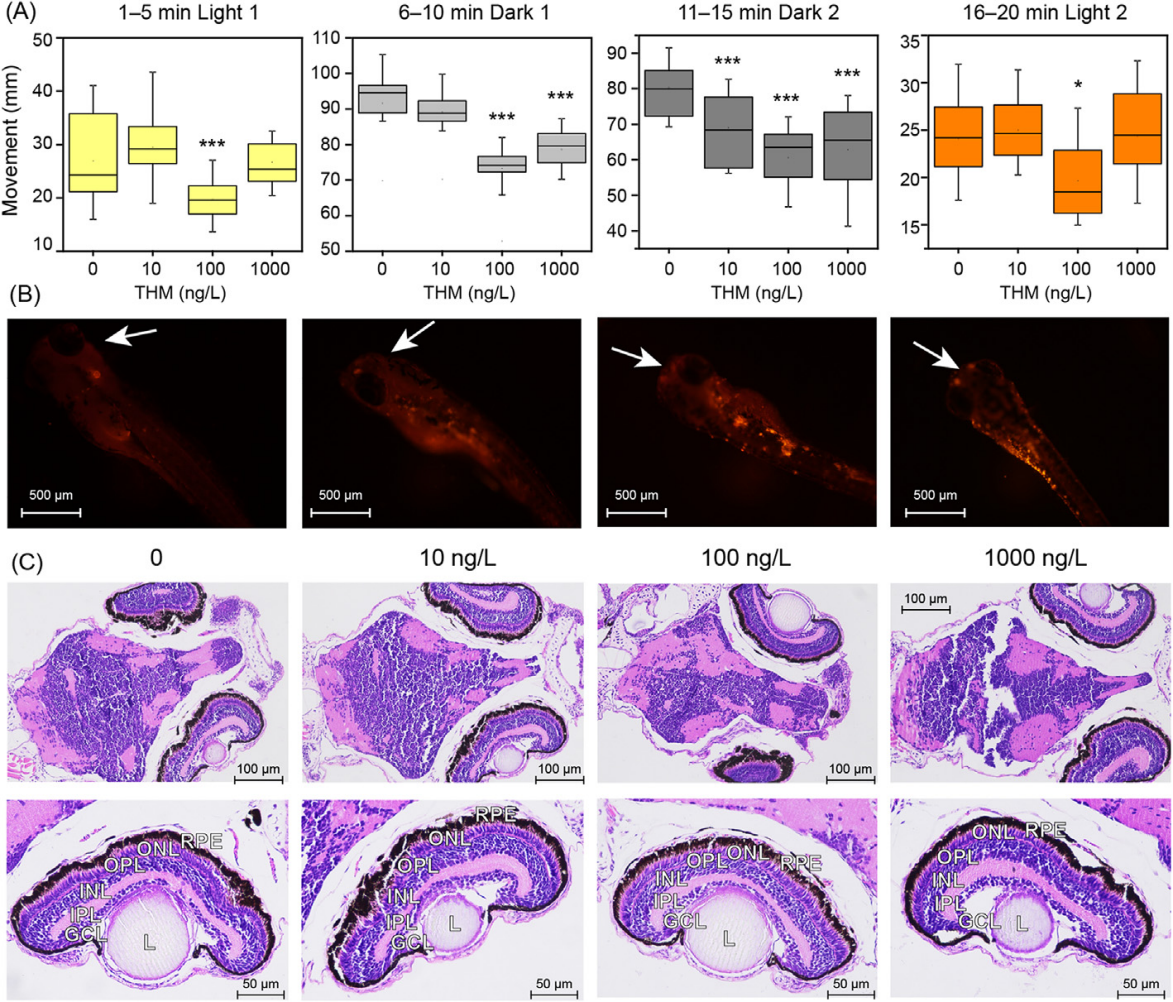
(A) Photomotor response of zebrafish larvae at 120 hpf. (B) Fluorescent microscopic images of the larvae at 120 hpf, with white arrow indicating fluorescence around the eyes. (C) Histological changes in the brain and retinal tissues of zebrafish larvae at 120 hpf. GCL, ganglion cell layer; INL, inner nuclear layer; IPL, inner plexiform layer; L, lens; ONL, outer nuclear cell; OPL, outer plexiform layer; REP, retinal pigmented epithelium.

Exposure to THM resulted in a significant reduction in zebrafish movement under both continuous light–dark transition stimulation and continuous dark periods (Fig. 1A). In comparison with the control group, even at concentrations as low as 10, 100, and 1000 ng/L, the movement in the second dark period was greatly reduced by 14.06%, 24.53%, and 21.64%, respectively. Among the gradient exposure concentrations, THM at 100 ng/L showed the strongest inhibition of locomotion, significantly reducing the average movement by 18.34%–26.52% in the entire test period.

As depicted in Fig. 1B and Fig. S8, the fluorescence intensity of the larvae gradually increased with the increment of THM concentrations. The average intensity in 10, 100, and 1000 ng/L groups were 1.28, 1.57, and 1.84 times that of the control group, with significant differences observed at 100 and 1000 ng/L (*p* < 0.05). Noticeably, the fluorescence intensity of the eye area changed distinctively, and the average intensity increased by 35.9%–83.2% after THM exposure, indicating the enhanced apoptosis of the cells in the eyes induced by THM.

As observed in the histological analysis (Fig. 1C), the brain tissue of zebrafish in the control group exhibited well-organized structures, with clear stratification between gray and white matter and dense cellularity in the granular layer. No notable histological alterations were detected after exposure to THM at 10 ng/L. However, at a concentration of 100 ng/L, a significant reduction in the number of granular layer cells was evident. This deterioration worsened at 1000 ng/L, where cells in the granular layer became markedly fewer, loosely arranged, or even absent. In addition to the brain, the eye region also showed pronounced histological changes. In the control group, retinal morphology was normal, with well-defined boundaries and properly aligned cells in the outer nuclear layer (ONL), inner nuclear layer (INL), and ganglion cell layer (GCL). In contrast, after exposure to 1000 ng/L THM, the GCL displayed a significant reduction in cell density.

Moreover, transcriptomic analysis revealed significant changes in the expressions of 53 genes associated with the neuronal and visual systems (Fig. S9). To validate the RNA-Seq results, the expressions of 5 representative genes (*elavl3*, *adra2b*, *gap43*, *opn1sw1* and *rho*) were quantified (Fig. S10). The expression of *elavl3* and *adra2b* were 2.01 and 1.89 folds of that in the control group, while the ratios of *gap43*, *opn1sw1* and *rho* were 0.43, 0.36 and 0.42, aligning with the RNA-Seq findings.

### 3.2 Transcriptomic alteration and its association with phenotypical changes

**Fig. 2:**
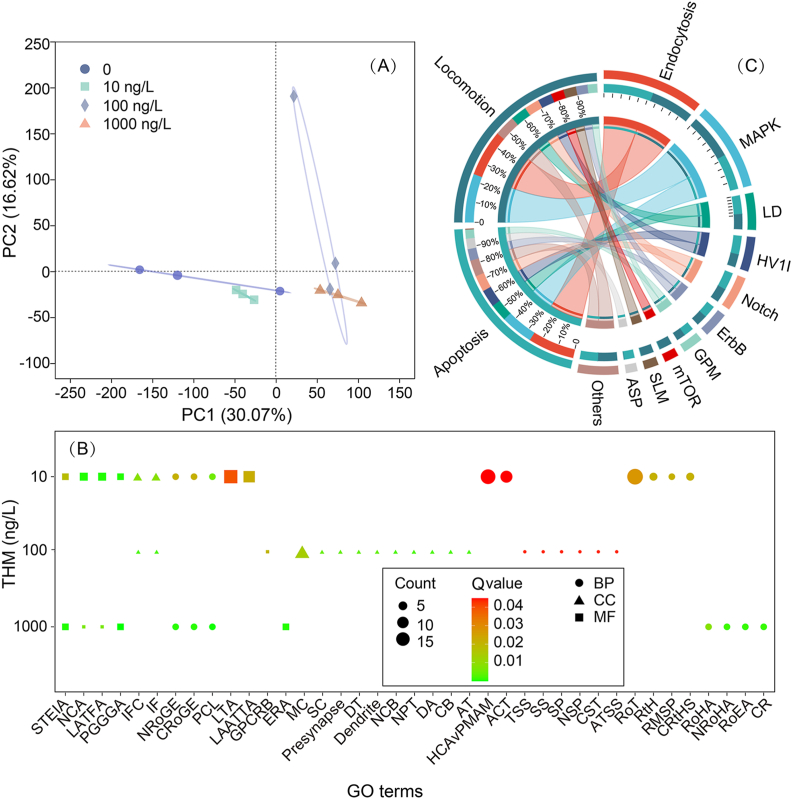
(A) Principal coordinate analysis (PCoA) of gene expression profiles. (B) GO terms of DEGs after exposure to THM. (C) Circos diagram illustrating the interaction of traits and enriched KEGG pathways. ACT, amino acid transport; ASP, Adipocytokine signaling pathway; AT, axon terminus; ATSS, anterograde trans-synaptic signaling; BP, biological processes; CB, cell body; CC, cellular components; CR, circadian rhythm; CRoGE, circadian regulation of gene expression; CRtHS, cellular response to hormone stimulus; CST, chemical synaptic transmission; DA, distal axon; DT, dendritic tree; ERA, endopeptidase regulator activity; ErbB, ErbB signaling pathway; GPCRB, G protein-coupled receptor binding; GPM, Glycerophospholipid metabolism; HCAvPMAM, homophilic cell adhesion via plasma membrane adhesion molecules; HSV1I, Herpes simplex virus 1 infection; IF, intermediate filament; IFC, intermediate filament cytoskeleton; LAATTA, L-amino acid transmembrane transporter activity; LATFA, ligand-activated transcription factor activity; LD, Lysine degradation; LTA, lipid transporter activity; MAPK, MAPK signaling pathway; MC, myosin complex; MF, molecular functions; mTOR, mTOR signaling pathway; NCA, nuclear receptor activity; NCB, neuronal cell body; Notch, Notch signaling pathway; NPT, neuron projection terminus; NRoGE, negative regulation of endopeptidase activity; NRoHA, negative regulation of hydrolase activity; NSP, neuropeptide signaling pathway; PCL, peptide cross-linking; PGGGA, protein-glutamine gamma-glutamyltransferase activity; RMSP, hormone-mediated signaling pathway; RoEA, regulation of endopeptidase activity; RoT, regulation of transport; RoHA, regulation of hydrolase activity; RtH, response to hormone; SLM, Sphingolipid metabolism; SP, sensory perception; SS, synaptic signaling; STEIA, serine-type endopeptidase inhibitor activity; SC, somatodendritic compartment; TSS, trans-synaptic signaling.

The PCoA of gene expression profiles showed clear discrimination between the control and THM exposure groups (Fig. 2A), reflecting substantial transcriptomic changes. In general, we identified 19 (9 up-regulated and 10 down-regulated), 306 (302 up-regulated and 4 down-regulated), and 630 (606 up-regulated and 24 down-regulated) significant DEGs following 10, 100, and 1000 ng/L THM exposure, respectively (Fig. S11). In addition, 39 significantly enriched GO terms were identified, many of which are related to the neuronal system, such as neuronal cell body, neuron projection terminus, sensory perception, and neuropeptide signaling pathway (Fig. 2B). Besides, terms associated with the visual system, such as G-protein-coupled receptor binding, were also enriched. Moreover, three hormone-related terms, including response to hormone, hormone-mediated signaling pathway, and cellular response to hormone stimulus, were significantly enriched.

The WGCNA was constructed to explore the relationships between DEGs and traits (locomotion and apoptosis), identifying 18 distinct modules (Fig. S12A). These modules were color-coded, with certain modules showing significant association with specific traits. For instance, the MEgrey60 module displayed a strong positive correlation with the apoptosis trait (*r* = 0.721, *p* = 0.0082), while the MEblack module exhibited a negative correlation with apoptosis (*r* = −0.661, *p* = 0.019) (Fig. S12B). Further KEGG pathway enrichment analysis of the hub genes in each trait showed that the endocytosis and MAPK signaling pathway contributed 26% and 18%, respectively, to apoptosis, while 22% and 23%, respectively, to locomotion. The other KEGG pathways contributed less than 10% individually (Fig. 2C).

### 3.3 Metabolomic changes and joint analysis with transcriptome

**Fig. 3:**
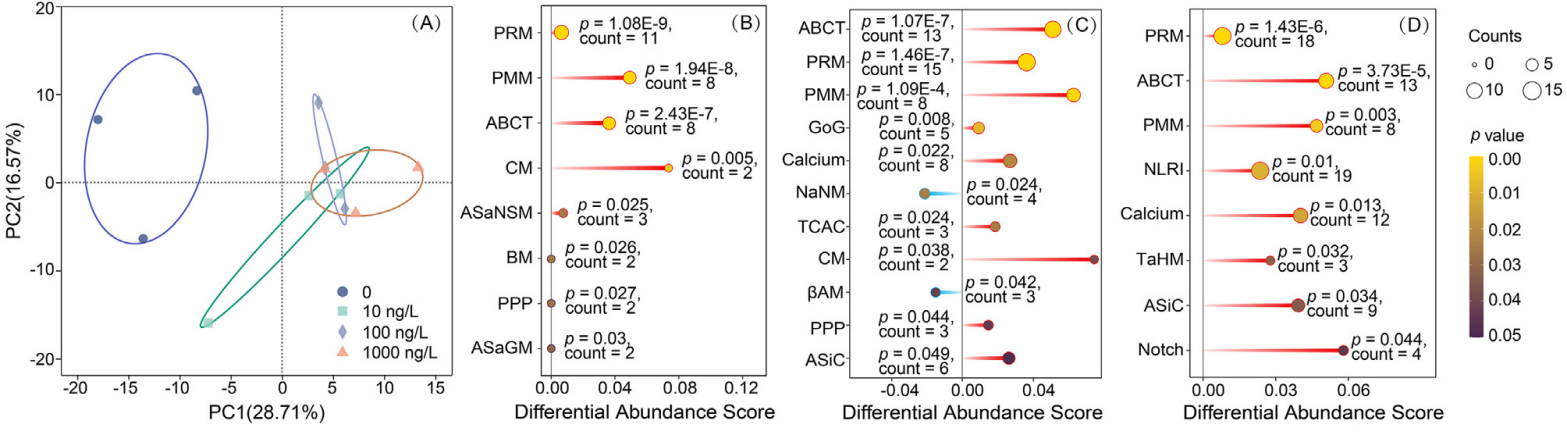
(A) PCoA of the metabolomics. (B–D) KEGG pathways of the DEGs and DCMs based on the joint analysis of transcriptomics and metabolomics at 10, 100 ng/L, and 1000 ng/L, respectively. The significance level was verified by *p* < 0.05. The tail in red or blue represents more up-regulated or down-regulated DEGs and DCMs, respectively. βAM, beta-Alanine metabolism; ABCT, ABC transporters; ASaGM, alanine, aspartate, and glutamate metabolism; ASaNSM, amino sugar and nucleotide sugar metabolism; BM, butanoate metabolism; Calcium, calcium signaling pathway; CM, caffeine metabolism; GoG, glycolysis or Gluconeogenesis; NaNM, nicotinate and nicotinamide metabolism; NLRI, neuroactive ligand-receptor interaction; PMM, pyrimidine metabolism; PPP, pentose phosphate pathway; PRM, purine metabolism; TaHM, taurine and hypotaurine metabolism; TCAC, TCA cycle.

PCoA of metabolites revealed a substantial distinction between the control and THM-treated groups, suggesting that distinctive metabolic changes occurred (Fig. 3A). In detail, a total of 78 (55 up-regulated and 23 down-regulated), 100 (77 up-regulated and 23 down-regulated), and 116 (85 up-regulated and 31 down-regulated) DCMs were identified following exposure to 10, 100, and 1000 ng/L of THM, respectively (Fig. S13). The KEGG pathway analysis highlighted 26 significantly enriched pathways after THM exposure (Fig. S14), with purine metabolism, ATP-binding cassette (ABC) transporters, and pyrimidine metabolism being the most significantly enriched pathways at all concentration groups. Integrative omics analysis also indicated these three pathways were the most significantly enriched pathways (Fig. 3B–D). In addition, pathways of calcium signaling pathway, neuroactive ligand–receptor interaction, nicotinate and nicotinamide metabolism were also significantly enriched in the groups of 100 and 1000 ng/L.

### 3.4 THM potentially binds with receptors and alters the content of neurotransmitters and hormones in zebrafish larvae

**Fig. 4:**
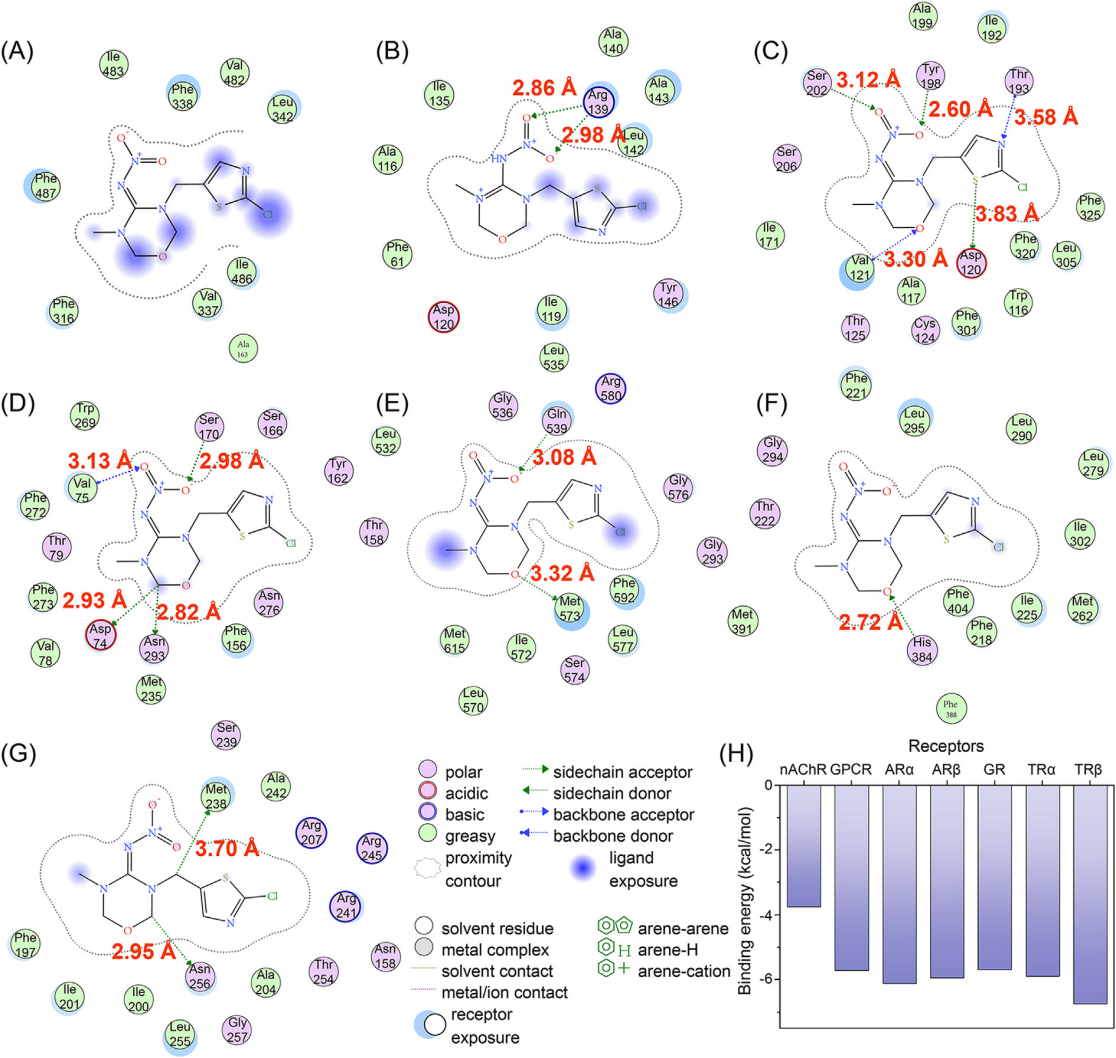
Predicted binding modes obtained from the docking simulation analysis of THM with target receptors in zebrafish. Two-dimensional views of the interaction of (A) THM-nAChR, (B) THM-GPCR, (C) THM-ARα, (D) THM-ARβ, (E) THM-GR, (F) THM-TRα, and (G) THM-TRβ. (H) Binding energies of THM with target receptors.

Molecular docking results revealed that the binding energy between THM and zebrafish nAChR was −3.75 kcal/mol, indicating a weak to medium binding affinity [31]. Additionally, THM formed strong hydrogen bond interactions with the residues Arg139 of GPCR, Ser202, Tyr198, Thr193, Val121, and Asp120 of ARα, Ser170, Val75, Asp74, and Asn293 of ARβ, Gln580 and Met573 of GR, His384 of TRα, and Met283 and Ile200 of TRβ (Fig. 4B−G). The binding energy between THM and GPCR, ARα, ARβ, GR, TRα, and TRβ ranged from −5.70 to −6.74 kcal/mol (Fig. 4H), also reflecting a weak to medium binding strength.

THM exposure significantly increased the activity of AChE by 14.07%–17.78% compared to the control group (Fig. S15A). This could be a compensatory response, as THM binding to nAChR may mimic ACh, leading to an increased demand for AChE to regulate ACh levels. As a result, the elevated AChE activity enhanced ACh hydrolysis, causing a significant reduction in ACh levels by 4.63%–18.83% (Fig. S15B), consistent with the metabolomic findings. In contrast, the content of ADR in zebrafish remained unchanged after THM exposure (Fig. S16A), but CORT contents increased significantly by 11.07% at 1000 ng/L THM (Fig. S16B). Additionally, the concentration of T3 significantly rose by 35.2%, while T4 and TSH significantly decreased by 27.01% and 8.38% at 1000 ng/L THM, respectively (Fig. S16C−E). T4 was the most sensitive hormone, with a significant decline detected at 100 ng/L. Moreover, the expression of several genes involved in nAChRs (*chrna4a*, *htrafa*, and *htr5aa*), GPCRs (*gpr139*, *gpr25*, *gpr174*), ARs (*adra1d* and *adrb3b*), GRs (*fkbp5*, *mmp21*, *mmp25a*, and *mmp16b*), and TRs (*rho*) were significantly affected by THM exposure at environmentally relevant concentrations (Fig. S17).

### 3.5 THM caused limited oxidative stress in zebrafish larvae

The results demonstrated that ROS content did not significantly increase after THM exposure (Fig. S18A). However, SOD and CAT were elevated by 8.60%–14.76% and 1.21%–20.10%, respectively, after THM exposure at concentrations ranging from 10 to 1000 ng/L, with significant increases occurred at 100 and 1000 ng/L (Fig. S18B, C). Furthermore, the expression of oxidative stress-related genes *cat* and *sod3b* showed slight increases with rising THM concentrations; however, these increases were not statistically significant (Fig. S18D).

## 4 Discussion

This study demonstrated that environmentally relevant concentrations of THM induce neurotoxicity in zebrafish, evidenced by alterations in locomotor behavior, histological changes of the brain tissue, and the expressions of relevant genes. Generally, neurotoxic effects became more pronounced with increasing THM concentrations. Even at the lowest concentration of 10 ng/L, both the photomotor response and AChE levels in zebrafish larvae were significantly affected. Other parameters did not show significant differences until concentrations reached 100 or 1000 ng/L. This disparity may be attributed to the varying sensitivities of the analytical methods employed. Consistent with our findings, previous research reported that THM significantly increased the overall wake activity in zebrafish at 100 μg/L [11]. Additionally, THM at concentrations of 50–50,000 ng/L was shown to alter the expression of genes related to behavior, CORT synthesis and neurodevelopment in zebrafish larvae [22]. In comparison, other neonicotinoid insecticides, including imidacloprid (45 μg/L), clothianidin (1–100 μM) and dinotefuran (1–100 μM) were found to significantly affect the dark-induced locomotion [32,33]. Although, except for zebrafish, THM exposure also caused brain histological changes to rats [34].

In addition to its direct effects on the neuronal system, THM also impaired visual processes and indirectly damaged the neuronal system of bees at 2.7 ng/L diet, a concentration similar to field residue levels [35]. In this study, the significant apoptosis observed in zebrafish, particularly around the eyes, is considered a key factor contributing to the behavioral change. The retina, as an extension of the central nervous system (CNS), plays a critical role in converting visual information into electrical signals and transmitting them to the brain [36]. Disruptions in visual structure can lead to neurological disorders, resulting in altered visually mediated behaviors [37]. Similarly, imidacloprid exposure at 10 and 100 μg/L caused behavioral changes, which were linked to disrupted opsin expression and impaired phototransduction in the zebrafish retina [38].

**Fig. 5:**
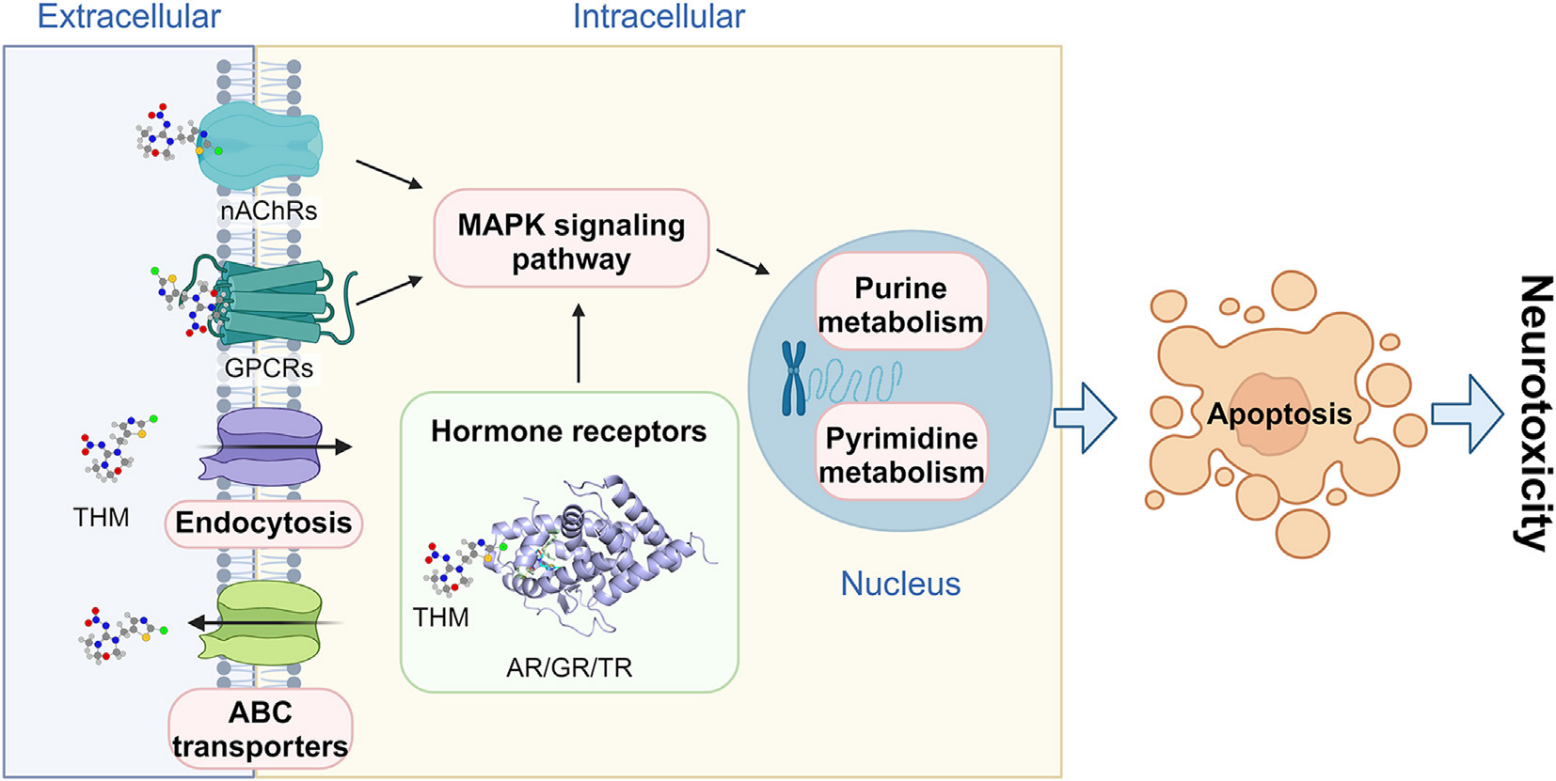
Proposed mechanisms for neurotoxicity induced by THM at environmentally relevant concentrations.

Based on integrative analyses involving multi-omics, molecular docking, and ELISA, we propose a mechanism pathway for THM-induced neurotoxicity (Fig. 5). Although THM was originally designed to selectively bind to the nAChR in insects [9], our findings suggest it also interreacts with the nAChR in zebrafish, as evidenced by molecular docking and ACh quantification results. Despite its low affinity for vertebrate nAChR, THM still affected the neurobehavior of larval fish [14]. Furthermore, THM may accumulate in the cytoplasm, facilitated by endocytosis and G-protein coupled receptors. This cytoplasmic accumulation enables THM to interact with multiple hormone receptors, including ARs, TRs, and GRs, which are primarily located in the cytoplasm [39]. The molecular docking results and the enrichment of hormone-related GO terms further substantiate this mechanism. The collective binding of THM with multiple receptors likely regulates the receptor-activated MAPK signaling pathway, influencing downstream purine and pyrimidine metabolism within the nucleus. This ultimately triggers cell apoptosis, contributing to THM's neurotoxicity.

Similar to our findings, a study on fipronil exposure at 40 μg/L also revealed neurotoxic effects in zebrafish, with enriched KEGG pathways including MAPK, calcium signaling, neuroactive ligand–receptor interaction, purine metabolism, and endocytosis [40]. THM appears to induce neurotoxicity through both extracellular and intracellular routines. nAChR is a transmembrane receptor that directly transmits signals such as neonicotinoid insecticides, leading to CNS dysfunction, which is fatal for insects [41]. Our molecular docking analysis revealed that the binding energy between THM and zebrafish nAChR was −3.75 kcal/mol, lower than the binding energies of another two neonicotinoid insecticides, imidacloprid and thiacloprid, to nAChRs of cockroach and honeybee (−6.07 to −7.06 kcal/mol) [42]. Despite this, THM significantly increased AChE activity, indicating its potential to disrupt the neuronal system. More importantly, nAChR can activate the MAPK pathway, leading to either cell division or apoptosis [43]. GPCRs are integral membrane proteins that play a vital role in transmitting extracellular signals like hormones and neurotransmitters into intracellular responses, affecting processes such as vision, olfaction, and taste [44]. The MAPK is a primary GPCR-induced intracellular signaling pathway that triggers downstream molecular events [45]. Overall, THM likely activates the MAPK signaling pathway through both nAChRs and GPCRs, leading to metabolic disturbances and neurotoxicity.

Besides, the relatively small molecular weight of THM (291.7 Da) suggests that it can enter the cytoplasm and exert effects through multiple pathways. Endocytosis, a fundamental process by which eukaryotic cells internalize macromolecules and proteins [46], plays a role in the uptake of drugs, nanoparticles, and viruses. In contrast, ABC transporters, membrane proteins involved in the efflux of toxic compounds and their metabolites [47], regulate the intracellular concentration of THM. Overall, after the combination effects of influx and efflux, some THM likely enter the cytoplasm and bind with various hormone receptors, disrupting cellular function. The activation of endocytosis is linked to the regulation of hormone receptors such as ARs, GRs and TRs in the CNS [[48], [49], [50]]. When THM binds to these receptors, the balance of related hormones might be disrupted. In this study, we observed a significant increase in CORT and T3 levels, alongside a decrease in T4 and TSH levels. The reduction in T4 and TSH levels may result from negative feedback in response to elevated T3 [51]. Disruptions in thyroid hormone levels during larval development are known to impair CNS development and can even affect the behavior of the next generation [52]. Moreover, the binding of THM to GRs can influence CORT levels [53], as demonstrated by the significant elevation of CORT observed in the THM-exposure groups. As the final product of the hypothalamic-pituitary-interrenal (HPI) axis, CORT plays a key role in stress response and can alter fish behavior [54]. Consistent with our findings, previous studies also reported elevated CORT levels in adult zebrafish exposed to THM at 1000 μg/L, while no significant changes were observed at lower concentrations (0.1 and 10 μg/L) [55]. This discrepancy might be explained by the higher sensitivity of the larval stage compared to the adult stage.

After binding to ARs, GRs and TRs, THM likely influences the MAPK signaling pathway. Previous studies have shown that the MAPK signaling pathway can be activated by thyroid hormones, which respond to shifts in homeostasis and environmental conditions [56]. Thus, THM binding to TRs may disrupt the balance of thyroid hormones in the organism, leading to MAPK signaling pathway activation [17]. Furthermore, the activated MAPK signaling pathway has also been linked to interactions with GRs, with research suggesting a connection to depressive disorders [57]. Therefore, the binding of THM to these hormone receptors likely triggers a cascade of downstream metabolic disturbances.

Purines and pyrimidine metabolism emerged as the primary altered pathways in both transcriptomic and metabolomic analyses, highlighting their critical involvement in THM-induced neurotoxicity. The activity of carbamoyl phosphate synthetase, a key enzyme in the *de novo* pyrimidine biosynthesis, is notably regulated by MAPK-mediated phosphorylation [58]. Similarly, the MAPK signaling pathway has been implicated in the regulation of purine metabolic enzymes [59]. Therefore, it is reasonable to conclude that the MAPK signaling pathway plays a central role in modulating these two metabolic pathways in zebrafish. Purines and pyrimidine are essential components of nucleotides, which serve as the building blocks of DNA and RNA [60]. Dysregulation of these pathways can negatively impact energy production, cell proliferation, and various cellular functions [60,61]. As a result, the dysregulation of purine and pyrimidine metabolism is likely a contributing factor to the observed apoptosis in zebrafish, ultimately leading to neurotoxicity. The disruption of these metabolic pathways might also pose negative effects to other aquatic organisms. For example, acetamiprid induced toxic effects on *Xenopus laevis* tadpoles by disrupting the purine metabolic pathway [62].

In addition, the activation of the MAPK pathway by environmental toxins has been associated with oxidative stress in the CNS [63]. However, the levels of ROS in zebrafish did not significantly increase, suggesting that oxidative stress may not be the primary driver of the neurotoxicity induced by THM at environmentally relevant concentrations. This observation may be attributed to the effective clearance of ROS by antioxidant enzymes such as SOD and CAT in zebrafish [64]. Nonetheless, this equilibrium may be disrupted at elevated THM concentrations, as a previous study showed that THM significantly increased ROS and SOD content in zebrafish liver at 0.3 mg/L [16].

In summary, this study revealed the neurotoxicity of THM at environmentally relevant concentrations and explored its potential toxic mechanisms. While previous research has documented the negative influence of neonicotinoid insecticides on the bee population and birds in the field, evidence regarding their toxicity on aquatic organisms in realistic environments remains limited [65]. Existing studies pointed out that the acute and chronic toxicity of THM were negligible in surface water, with regulatory standards for neonicotinoid insecticides, such as imidacloprid, based on LC_50_ or the lowest observed effect concentration [66]. This study suggested that neurotoxicity may serve as a more sensitive toxicity endpoint for THM and should be prioritized to safeguard aquatic life. Future investigation should assess the ecological impacts of neonicotinoid insecticide in realistic settings from multiple aspects. For example, investigating how environmental factors, such as pH, temperature, and co-existing contaminants, influence THM toxicity. As reported recently, the interaction between aged microplastics and THM significantly impaired the heart rate and locomotion of zebrafish larvae [13]. Additionally, the effects of long-term, low-dose THM exposure on the zebrafish population warrant thorough explorations.

## 5 Conclusion

This study established that THM exhibits neurotoxicity in zebrafish at environmentally relevant concentrations, proposing that this effect is mediated through interactions with multiple receptors. Behavioral changes appeared to be linked to the injury of neuronal and visual systems, as confirmed by notable histological changes, apoptosis in the eyes, and many DEGs. We hypothesized that THM may interact with nAChRs on the membrane and multiple hormone receptors in the plasma, potentially altering the content of neurotransmitters and hormones like ACh, CORT, T3, T4, and TSH. This interaction may subsequently activate the MAPK signaling pathway, which could impact purine and pyrimidine metabolism within the cell nucleus, ultimately leading to cell apoptosis. The findings underscore the critical need for assessing the adverse effects of THM in the aquatic system based on realistic conditions and emphasize the urgency of developing strategies to mitigate THM levels in the environment.

## CRediT authorship contribution statement

**Xiuwen Li:** Writing – original draft, Methodology, Data curation, Conceptualization. **Hanbing Zhao:** Validation, Formal analysis. **Minjuan Gong:** Software, Formal analysis. **Feng Zhang:** Supervision. **Shengnan Liu:** Visualization, Software. **Zepeng Zhang:** Visualization, Software. **Yide He:** Writing – review & editing. **Henner Hollert:** Writing – review & editing. **Xiaowei Zhang:** Writing – review & editing, Methodology. **Wei Shi:** Writing – review & editing. **Qing Zhou:** Writing – review & editing, Funding acquisition. **Aimin Li:** Supervision, Project administration, Funding acquisition. **Peng Shi:** Writing – review & editing, Supervision, Methodology, Funding acquisition.

## Declaration of competing interest

The authors declare no conflicts of interest.
