# Supplementary material for: Thiamethoxam at environmentally relevant concentrations induces neurotoxicity in zebrafish larvae through binding with multiple receptors
Source: Eco Environ Health. 2025 Jan 17;4(1):100133. doi: 10.1016/j.eehl.2024.12.002 (PMC11872479; doi:10.1016/j.eehl.2024.12.002)
Supplement: Multimedia component 1 [file mmc1.docx]

**Supporting Information**

**Thiamethoxam at environmentally relevant concentrations induces neurotoxicity in zebrafish larvae through binding with multiple receptors**

Xiuwen Li^a^, Hanbing Zhao^a^, Minjuan Gong^a^, Feng Zhang^b^, Shengnan Liu^a^, Zepeng Zhang^a^, Yide He^c^, Henner Hollert^d, e^, Xiaowei Zhang^a^, Wei Shi^a^, Qing Zhou^a^, Aimin Li^a^, Peng Shi^a*^

^a^ State Key Laboratory of Pollution Control and Resource Reuse, School of Environment, Nanjing University, Nanjing 210023, China.

^b^ Key Laboratory of Mesoscopic Chemistry of Ministry of Education (MOE), School of Chemistry and Chemical Engineering, Nanjing University, Nanjing 210023, China

^c^ School of Environmental Science and Engineering, Nanjing Tech University, Nanjing 211816, China.

^d^ Department Evolutionary Ecology & Environmental Toxicology, Goethe University, Frankfurt 60438, Germany.

^e^ Department Environmental Media Related Ecotoxicology, Fraunhofer Institute for Molecular Biology and Applied Ecology IME, Schmallenberg 57392, Germany.

***Corresponding author:**

**Email:** shipeng@nju.edu.cn (Shi Peng)

**Total Pages: 39**

**Number of texts: 3**

**Number of tables: 6**

**Number of figures: 18**

**Texts**

**Text S1.** Chemical analysis of thiamethoxam (THM) in exposure solutions.

**Text S2.** Homology modeling and docking analysis

**Text S3.** The chromatographic and MS conditions for metabolomics analysis

**Tables**

**Table S1.** Detected concentration of THM in exposure solutions after each renewal.

**Table S2.** The quality of homology modeling.

**Table S3.** Descriptions of genes involved in the neuronal and visual system.

**Table S4.** Primer pairs used in qRT-PCR analysis.

**Table S5.** Thermal procedures of qRT-PCR.

**Table S6.** The gradient of mobile phase for the instrumental analysis by UPLC-TripleTOF system.

**Figures**

**Fig. S1.** The modeling quality of nAChR-zebrafish.

**Fig. S2.** The modeling quality of GPCR-zebrafish.

**Fig. S3.** The modeling quality of ARα-zebrafish.

**Fig. S4.** The modeling quality of ARβ-zebrafish.

**Fig. S5.** The modeling quality of GR-zebrafish.

**Fig. S6.** The modeling quality of TRα-zebrafish.

**Fig. S7.** The modeling quality of TRβ-zebrafish.

**Fig. S8.** The fluorescence intensity quantified by Image J.

**Fig. S9.** Principal coordinate analysis (PCoA) of DEGs involved in the neuronal system (A) and visual system(B). Heatmaps of DEGs involved in the neuronal system (C) and visual system (D).

**Fig. S10.** PCR results of selected genes involved in the neuronal and visual systems. The asterisks (*) represent significant differences between the control group and the experimental groups (*, *p* < 0.05).

**Fig. S11.** Venn diagram for significantly up-regulated and down-regulated DEGs after exposure to THM at 10, 100, and 1000 ng/L.

**Fig. S12.** WGCNA module identification and correlation analysis. The cluster dendrogram of DEGs with dissimilarity based on the topological overlap and assigned module colors (A). Correlation of the identified modules with different traits (B).

**Fig. S13.** Venn diagram for significantly up-regulated and down-regulated DCMs.

**Fig. S14.** KEGG pathways of the DCMs after exposure to THM.

**Fig. S15.** Alteration on biomarkers in the neuronal system after THM exposure. (A) AChE: acetylcholinesterase, (B) ACh: acetylcholine. The asterisks (*) represent significant differences between the control and the experimental groups (*, *p* < 0.05, **, *p* < 0.01, and ***, *p* < 0.001).

**Fig. S16.** Alteration on hormones after THM exposure. (A) ADR: adrenaline, (B) CORT: cortisol, (C) T3: triiodothyronine, (D) T4: thyroxine, (E) TSH: thyroid-stimulating hormone. The asterisks (*) represent significant differences between the control and the experimental groups (*, *p* < 0.05, **, *p* < 0.01, and ***, *p* < 0.001).

**Fig. S17.** Expression of genes involved in nAChRs, GPCRs, ARs, GRs, and TRs after THM exposure.

**Fig. S18.** Alteration on biomarkers and related genes in the antioxidant system after THM exposure. (A) ROS: reactive oxygen species, (B) CAT: catalase, (C) SOD: superoxide dismutase, and (D) Gene expression of *cat* and *sod3b*. The asterisks (*) represent significant differences between the control and the experimental groups (*, *p* < 0.05 and **, *p* < 0.01).

## **Text S1.** Chemical analysis of thiamethoxam (THM) in exposure solutions.

Actual THM concentrations in the exposure solutions were measured after renewal of the water solution. After collection, the exposure solutions were filtered through a 0.45 μm glass-fiber filter (Millipore, U.S.) before spiking with 10 ng/L of imidacloprid-d4 as the internal standard. Subsequently, 50 mL of each exposure solution was loaded onto a Waters Oasis HLB cartridge (500 mg, 6 mL, Milford, MA, U.S.), which was preconditioned with 5 mL of methanol and water, respectively. Then, the cartridge was rinsed with 5 mL of water and vacuum dried for 5 min, and the target compounds were eluted with 4 mL of acetonitrile, methanol, dichloromethane, and ethyl acetate, respectively. Finally, the eluates were evaporated to dryness under a gentle nitrogen stream at 35 °C and reconstituted with 1 mL of methanol. The nylon filters were applied to remove suspended solids in the samples before instrumental analysis.

Detection of THM was performed on an Ultra-high Performance Liquid Chromatography & Tandem Quadrupole Mass Spectrometry (UPLC-Class & Xevo TQ-MS, Waters, U.S.) with a C_18_ column (1.7 μm, 2.1 mm × 100 mm, Waters, U.S.) at 35 °C according to the method in our previous study ^1^. The mobile phases were ultrapure water (mobile phase A) and methanol (mobile phase B). Five microliters of each concentrated sample were injected and separated using a gradient elution program with a flow rate of 0.3 mL/min. The MS program was conducted with multiple reaction monitoring in a positive electrospray ionization mode. The precursor ion, quantification ion, and confirmation ion of THM were 291.8, 211, and 131.9 *m/z*, respectively. The cone energy was 10 V, and the collision energy was 24/14 eV. The recoveries of THM in water ranged between 94% and 106%. The method detection limit (MDL) and method quantitation limits (MQL) for each analyte were determined as the lowest concentrations that yielded signal-to-noise ratios of 3 and 10, respectively. In this study, the MDL and MQL for THM were 4.0 ng/L and 16.3 ng/L, respectively.

## **Text S2.** Homology modeling and docking analysis

To construct the structural models of nicotinic acetylcholine receptor (nAChR), G-protein-coupled receptor (GPCR), adrenoceptor alpha (ARα), ARβ, aryl hydrocarbon receptor (AhR), glucocorticoid receptor (GR), thyroid hormone receptor alpha (TRα), and TRβ in zebrafish, the corresponding amino acid sequences were obtained from the BLAST server (http://blast.ncbi.nlm.nih.gov) for searching protein templates. The GenBank IDs of these receptors were AAI71405.1, NP_001007200.2, NP_001311383.1, NP_001121807.1, NP_001018547.2, NP_571471.1, and NP_571415.1, respectively. Homology modeling of the peptide chains was performed using the modeller 10.1 mounting package (https://salilab.org/modeller/). The binding modes and binding energies of THM to the zebrafish receptors were generated by AutoDock Tools. The docked active site was defined as the location of the ligand in the template protein. THM was optimized for energy minimization using AutoDock Vina, and AutoDock Tools 1.5.6, to dock into the active pocket of these receptors. Finally, a docking illustration was generated, and the protein-ligand complex was analyzed with PyMOL v.1.3.

## **Text S3.** The chromatographic and MS conditions for metabolomics analysis

10 μL of sample was injected and separated through a HSS T3 column (100 mm × 2.1 mm i.d., 1.8 μm) before entering mass spectrometry detection. The mobile phases consisted of 0.1% formic acid in water:acetonitrile (95:5, v/v) (solvent A) and 0.1% formic acid in acetonitrile:isopropanol:water (47.5:47.5:5, v/v/v) (solvent B). The solvent gradients are presented in Table S6. The flow rate was 0.4 mL/min, and the column temperature was maintained at 40 °C. The mass conditions were set as follows: Source temperature at 550 °C; curtain gas (CUR) at 30 psi; both Ion Source Gas1 and Gas2 at 50 psi; ion-spray voltage floating (ISVF) at −4000 V in negative mode and 5000 V in positive mode, respectively; declustering potential at 80 V; collision energy (CE), 20–60 eV rolling for MS/MS. Data acquisition was performed in Information Dependent Acquisition (IDA) mode. The detection was carried out over a mass range of 50–1000 m/z.

## **Table S1.** Detected concentration of THM in exposure solutions before each renewal.

| **Nominal**  **concentrations (ng/L)** | **Detected concentrations (ng/L)** | | | | |
| --- | --- | --- | --- | --- | --- |
|  | **0 h** | **24 h** | **48 h** | **72 h** | **96 h** |
| 10 | 11.3 ± 0.8 | 9.7 ± 1.4 | 8.7 ± 1.2 | 9.3 ± 0.5 | 8.5 ± 0.9 |
| 100 | 95.6 ± 1.5 | 83.2 ± 0.8 | 81.9 ± 1.9 | 88.9 ± 2.1 | 86.9 ± 1.5 |
| 1000 | 983.5 ± 1.6 | 863.4 ± 1.6 | 844.8 ± 2.6 | 889.7 ± 1.4 | 921.5 ± 3.8 |

## **Table S2.** The quality of homology modeling.

| **Receptor/Item** | **nAChR** | **GPCR** | **ARα** | **ARβ** | **GR** | **TRα** | **TRβ** |
| --- | --- | --- | --- | --- | --- | --- | --- |
| Residues in most favoured regions [A,B,L] | 84.3% | 89.0% | 86.2% | 91.9% | 87.7% | 94.0% | 94.3% |
| Residues in additional allowed regions [a,b,l,p] | 13.3% | 9.6% | 12.8% | 6.8% | 10.4% | 5.2% | 5.7% |
| Residues in generously allowed regions [~a,~b,~l,~p] | 1.2% | 1.1% | 0.4% | 0.7% | 1.8% | 0.9% | 0.0% |
| Residues in disallowed regions | 1.2% | 0.4% | 0.7% | 0.7% | 0.0% | 0.0% | 0.0% |
| Acceptable amino acid conformations | 98.8% | 100.0% | 99.3% | 99.3% | 100.0% | 100.0% | 100.0% |

## **Table S3.** Descriptions of genes involved in the neuronal and visual system.

| **Gene name** | **Description** |
| --- | --- |
| *elavl3* ^2^ | Predicted to enable RNA binding activity. Acts upstream of or within the regulation of neurogenesis. Predicted to be part of the ribonucleoprotein complex. Is expressed in several structures, including nervous system; neural plate; neural tube; neuronal stem cell; and presumptive structure. |
| *nkx2.2a* ^2^ | Predicted to enable DNA-binding transcription factor activity, RNA polymerase II-specific and RNA polymerase II cis-regulatory region sequence-specific DNA binding activity. Involved in positive regulation of DNA-templated transcription. Acts upstream of or within endocrine pancreas development; floor plate formation; and neurogenesis. Predicted to be active in the nucleus. Is expressed in several structures, including digestive system; nervous system; neural keel; neural rod; and neural tube. |
| *gap43* ^2^ | Predicted to enable calmodulin binding activity and phospholipid binding activity. Acts upstream of or within axon regeneration and tissue regeneration. Predicted to be located in filopodium membrane; growth cone membrane; and synapse. Predicted to be active in cytoplasm; plasma membrane; and postsynaptic density. Is expressed in nervous system; neural tube; and trigeminal placode. |
| *neurog1* ^2^ | Predicted to enable DNA-binding transcription factor activity, RNA polymerase II-specific; E-box binding activity; and chromatin binding activity. Involved in generation of neurons; hindbrain morphogenesis; and peripheral nervous system development. Acts upstream of or within several processes, including negative regulation of neurogenesis; nervous system development; and olfactory placode development. Predicted to be active in nucleus. Is expressed in several structures, including ectoderm; nervous system; neural keel; neural plate; and neural tube. |
| *syn2a* ^2^ | Predicted to enable ATP binding activity. Predicted to be involved in neurotransmitter secretion. Predicted to be located in synaptic vesicle. Predicted to be active in synaptic vesicle membrane. Is expressed in DEL; nervous system; spinal cord neural tube; and yolk syncytial layer. |
| *gfap* ^2^ | Predicted to be a structural constituent of the cytoskeleton. Predicted to be involved in intermediate filament organization and regulation of chaperone-mediated autophagy. Located in the cytoplasm and type III intermediate filament. Is expressed in several structures, including anterior neural keel; nervous system; neural tube; neuronal stem cell; and optic vesicle. |
| *mbpb* ^2^ | Predicted to be a structural constituent of myelin sheath. Located in the myelin sheath and plasma membrane. Is expressed in nervous system and polster. Human ortholog(s) of this gene implicated in multiple sclerosis. |
| *rpl13a ^2^* | Predicted to enable mRNA binding activity. Predicted to be a structural constituent of ribosome. Predicted to be involved in negative regulation of translation. Predicted to act upstream of or within translation. Predicted to be part of cytosolic large ribosomal subunit. Predicted to be active in ribosome. Is expressed in several structures, including blastomere; heart; liver; musculature system; and pleuroperitoneal region. |
| *grm6a* ^3^ | Predicted to enable group III metabotropic glutamate receptor activity. Predicted to be involved in G protein-coupled glutamate receptor signaling pathway and regulation of glutamatergic synaptic transmission. Predicted to act upstream of or within adenylate cyclase-inhibiting G protein-coupled glutamate receptor signaling pathway. Predicted to be located in plasma membrane. Is expressed in brain and retinal neural layer. |
| *drd1b* ^3^ | Predicted to enable G protein-coupled receptor activity and dopamine neurotransmitter receptor activity, coupled via Gs. Predicted to be involved in adenylate cyclase-activating adrenergic receptor signaling pathway and dopamine receptor signaling pathway. Predicted to act upstream of or within adenylate cyclase-activating G protein-coupled receptor signaling pathway and vasodilation. Predicted to be located in membrane. Is expressed in nervous system. |
| *adrb2a* ^3^ | Predicted to enable beta2-adrenergic receptor activity and norepinephrine binding activity. Acts upstream of or within pigmentation and regulation of heart rate. Predicted to be located in membrane. Is expressed in several structures, including blood; digestive system; musculature system; nervous system; and pleuroperitoneal region. |
| *adra2b* ^3^ | Enables alpha2-adrenergic receptor activity. Involved in adenylate cyclase-inhibiting adrenergic receptor signaling pathway. Predicted to be located in plasma membrane. Is expressed in several structures, including digestive system; eye; heart; integument; and pleuroperitoneal region. |
| *npy8br* ^3^ | Enables neuropeptide binding activity. Predicted to be involved in G protein-coupled receptor signaling pathway. Predicted to act upstream of or within signal transduction. Predicted to be located in membrane. Predicted to be active in neuron projection and plasma membrane. Is expressed in several structures, including brain; digestive system; hatching gland; immune system; and otic vesicle. |
| *gria3a* ^3^ | Predicted to enable AMPA glutamate receptor activity and transmitter-gated monoatomic ion channel activity involved in regulation of postsynaptic membrane potential. Predicted to be involved in glutamatergic synaptic transmission and modulation of chemical synaptic transmission. Predicted to act upstream of or within monoatomic ion transport. Predicted to be located in postsynaptic membrane. Predicted to be part of AMPA glutamate receptor complex. Predicted to be active in dendritic spine and postsynaptic density membrane. Is expressed in nervous system. |
| *htr1aa* ^4^ | Predicted to enable G protein-coupled serotonin receptor activity; neurotransmitter receptor activity; and serotonin binding activity. Predicted to be involved in G protein-coupled receptor signaling pathway, coupled to cyclic nucleotide second messenger; adenylate cyclase-inhibiting serotonin receptor signaling pathway; and chemical synaptic transmission. Predicted to act upstream of or within several processes, including regulation of behavior; regulation of hormone secretion; and regulation of vasoconstriction. Predicted to be located in membrane. Predicted to be active in dendrite. Is expressed in male organism; nervous system; and pharynx. |
| *htr1ab* ^4^ | Predicted to enable G protein-coupled serotonin receptor activity; neurotransmitter receptor activity; and serotonin binding activity. Acts upstream of or within spinal cord motor neuron differentiation. Predicted to be located in membrane. Predicted to be active in dendrite. Is expressed in brain; pharynx; retina; and spinal cord. |
| *htr1b* ^4^ | Predicted to enable G protein-coupled serotonin receptor activity and neurotransmitter receptor activity. Predicted to be involved in G protein-coupled receptor signaling pathway, coupled to cyclic nucleotide second messenger; adenylate cyclase-inhibiting serotonin receptor signaling pathway; and chemical synaptic transmission. Predicted to act upstream of or within several processes, including bone remodeling; regulation of behavior; and vasoconstriction. Predicted to be located in plasma membrane. Predicted to be active in dendrite. Is expressed in central nervous system; male organism; pharynx; and retina. |
| *nes* ^4^ | Predicted to enable intermediate filament binding activity. Involved in brain development; embryonic camera-type eye development; and negative regulation of apoptotic process. Acts upstream of or within cranial nerve development. Predicted to be located in intermediate filament. Is expressed in several structures, including adaxial cell; gut epithelium; nervous system; neural crest; and neuronal stem cell. |
| *nr4a2b* ^4^ | Predicted to enable DNA-binding transcription factor activity, RNA polymerase II-specific; RNA polymerase II cis-regulatory region sequence-specific DNA binding activity; and nuclear glucocorticoid receptor binding activity. Acts upstream of or within dopaminergic neuron differentiation; forebrain neuron development; and neutrophil homeostasis. Predicted to be located in cytoplasm. Predicted to be part of transcription regulator complex. Predicted to be active in nucleus. Is expressed in basal plate midbrain region; blood; cranial neural crest; nervous system; and neuroblasts. |
| *bdnf* ^4^ | Predicted to enable growth factor activity and nerve growth factor receptor binding activity. Acts upstream of or within circadian behavior; multicellular organismal response to stress; and nervous system development. Predicted to be active in several cellular components, including axon; dendrite; and synaptic vesicle. Is expressed in several structures, including blastoderm; digestive system; kidney; nervous system; and oral region. |
| *th2* ^4^ | Enables tryptophan 5-monooxygenase activity. Acts upstream of or within dopamine biosynthetic process and serotonin biosynthetic process. Predicted to be active in axon; cytoplasm; and perikaryon. Is expressed in female organism; head; kidney; liver; and nervous system. |
| *dnmt3aa* ^5^ | Predicted to enable DNA (cytosine-5-)-methyltransferase activity; DNA binding activity; and metal ion binding activity. Acts upstream of or within response to temperature stimulus and swimming behavior. Predicted to be active in cytoplasm and nucleus. Is expressed in several structures, including digestive system; nervous system; pleuroperitoneal region; spinal cord neural tube; and swim bladder bud. |
| *drd4b* ^5^ | Predicted to enable G protein-coupled serotonin receptor activity; neurotransmitter receptor activity; and serotonin binding activity. Predicted to be involved in G protein-coupled receptor signaling pathway, coupled to cyclic nucleotide second messenger; adenylate cyclase-inhibiting serotonin receptor signaling pathway; and chemical synaptic transmission. Predicted to act upstream of or within adenylate cyclase-inhibiting dopamine receptor signaling pathway. Predicted to be located in membrane. Predicted to be active in dendrite. Is expressed in endoderm; nervous system; neural tube; and pharyngeal arch 3-7 skeleton. |
| *dnmt1* ^5^ | Enables DNA (cytosine-5-)-methyltransferase activity, acting on CpG substrates. Involved in DNA methylation and negative regulation of gene expression. Acts upstream of or within several processes, including camera-type eye development; digestive system development; and hemopoiesis. Predicted to be active in the nucleus. Is expressed in several structures, including digestive system; immature eye; nervous system; neural tube; and pectoral fin. |
| *neurod2* ^6^ | Predicted to enable DNA-binding transcription factor activity, RNA polymerase II-specific and E-box binding activity. Predicted to be involved in axon development; positive regulation of transcription by RNA polymerase II; and sensory organ development. Predicted to act upstream of or within several processes, including regulation of DNA-templated transcription; regulation of neuron differentiation; and regulation of synapse maturation. Predicted to be active in the nucleus. Is expressed in granule cell. |
| *neurod6b* ^6^ | Predicted to enable DNA-binding transcription factor activity, RNA polymerase II-specific and E-box binding activity. Predicted to be involved in axon development; positive regulation of transcription by RNA polymerase II; and sensory organ development. Predicted to act upstream of or within cell differentiation; nervous system development; and regulation of DNA-templated transcription. Predicted to be active in nucleus. Is expressed in brain. |
| *foxg1b* ^6^ | Predicted to enable sequence-specific double-stranded DNA binding activity. Predicted to be involved in regulation of transcription by RNA polymerase II. Predicted to act upstream of or within regulation of DNA-templated transcription. Predicted to be active in the nucleus. Is expressed in anterior neural plate; immature eye; retina; and telencephalon. |
| *sox10* ^6^ | Predicted to enable DNA-binding transcription factor activity, RNA polymerase II-specific and RNA polymerase II cis-regulatory region sequence-specific DNA binding activity. Involved in olfactory bulb development. Acts upstream of or within with a positive effect on iridophore differentiation; melanocyte differentiation; and xanthophore differentiation. Acts upstream of or within several processes, including enteric nervous system development; glial cell differentiation; and inner ear development. Predicted to be active in the nucleus. Is expressed in several structures, including glioblast; head; iridoblast; nervous system; and neural crest. |
| *wnt1* ^6^ | Predicted to enable cytokine activity and frizzled binding activity. Acts upstream of or within brain development. Predicted to be located in the extracellular region. Predicted to be active in extracellular space. Is expressed in several structures, including central nervous system; midbrain hindbrain boundary neural keel; neural plate; neural rod; and neural tube. |
| *slc17a6b* ^6^ | Predicted to enable membrane potential driven uniporter activity; neurotransmitter transmembrane transporter activity; and salt transmembrane transporter activity. Involved in visual perception. Acts upstream of or within the chemical synaptic transmission. Predicted to be located in neuron projection and synaptic vesicle membrane. Predicted to be active in excitatory synapses. Is expressed in nervous system and trigeminal placode. |
| *slc17a6a* ^6^ | Predicted to enable membrane potential-driven uniporter activity; neurotransmitter transmembrane transporter activity; and salt transmembrane transporter activity. Predicted to be involved in several processes, including glutamatergic synaptic transmission; neurotransmitter loading into synaptic vesicle; and sodium-dependent phosphate transport. Predicted to act upstream of or within neurotransmitter transport; sodium ion transport; and transmembrane transport. Predicted to be located in neuron projection and synaptic vesicle membrane. Predicted to be active in excitatory synapse. Is expressed in central nervous system and neurons. |
| *manf* ^7^ | Acts upstream of or within dopaminergic neuron differentiation. Predicted to be active in endoplasmic reticulum and extracellular space. Is expressed in several structures, including axis; brain; hatching gland; pleuroperitoneal region; and sensory system. |
| *ache* ^7^ | Enables acetylcholinesterase activity. Acts upstream of or within several processes, including acetylcholine catabolic process; cellular response to methanol; and response to amphetamine. Predicted to be located in extracellular region; membrane; and synapse. Predicted to be active in extracellular space and plasma membrane. Is expressed in several structures, including female organism; male organism; muscle; nervous system; and neural tube. |
| *ambra1a* ^8^ | Predicted to enable ubiquitin ligase-substrate adaptor activity. Acts upstream of or within several processes, including autophagy; locomotory behavior; and skeletal muscle fiber development. Predicted to be located in several cellular components, including autophagosome; focal adhesion; and mitochondrion. Predicted to be part of Cul4-RING E3 ubiquitin ligase complex. Predicted to be active in nucleus. Is expressed in several structures, including blastodisc; oral cavity; otic vesicle; pleuroperitoneal region; and tail bud. |
| *ambra1b* ^8^ | Predicted to enable ubiquitin ligase-substrate adaptor activity. Acts upstream of or within chordate embryonic development; locomotory behavior; and skeletal muscle fiber development. Predicted to be located in several cellular components, including autophagosome; focal adhesion; and mitochondrion. Predicted to be part of Cul4-RING E3 ubiquitin ligase complex. Predicted to be active in the nucleus. Is expressed in several structures, including blastodisc; oral cavity; otic vesicle; pleuroperitoneal region; and tail bud. |
| *panx1a* ^9^ | Enables gap junction hemichannel activity and voltage-gated channel activity. Acts upstream of or within the dopamine receptor signaling pathway and visual perception. Located in a ruffle membrane. Is expressed in several structures, including heart; integument; liver; nervous system; and pleuroperitoneal region. |
| *panx1b* ^9^ | Enables voltage-gated channel activity and wide pore channel activity. Acts upstream of or within the detection of light stimulus involved in visual perception and swimming behavior. Located in cytoplasmic vesicle; perinuclear region of cytoplasm; and plasma membrane. Is expressed in several structures, including central nervous system; eye; heart; liver; and pleuroperitoneal region. |
| *opn1sw1* ^9^ | Predicted to enable G protein-coupled photoreceptor activity. Predicted to be involved in G protein-coupled receptor signaling pathway; cellular response to light stimulus; and phototransduction. Predicted to act upstream of or within signal transduction and visual perception. Predicted to be located in membrane. Predicted to be active in the photoreceptor outer segment. Is expressed in several structures, including digestive system; endocrine system; heart; photoreceptor cell; and visual system. |
| *opn1mw1* ^9^ | Predicted to enable G protein-coupled photoreceptor activity. Predicted to be involved in G protein-coupled receptor signaling pathway; cellular response to light stimulus; and phototransduction. Predicted to act upstream of or within signal transduction and visual perception. Located in the photoreceptor outer segment. Is expressed in several structures, including endocrine system; heart; integument; photoreceptor cell; and visual system. |
| *opn4xb* ^10^ | Enables photoreceptor activity. Acts upstream of or within the regulation of locomotor rhythm. Predicted to be located in the membrane. Is expressed in several structures, including endocrine system; heart; integument; liver; and visual system. |
| *rho* ^10^ | Enables G protein-coupled photoreceptor activity and retinal binding activity. Acts upstream of or within the absorption of visible light. Located in the photoreceptor outer segment. Is expressed in several structures, including digestive system; endocrine system; photoreceptor cell; presumptive neural retina; and visual system. |
| *rhol* ^10^ | Enables photoreceptor activity and retinal binding activity. Predicted to be involved in G protein-coupled receptor signaling pathway; cellular response to light stimulus; and phototransduction. Predicted to act upstream of or within signal transduction and visual perception. Predicted to be located in membrane. Predicted to be active in photoreceptor outer segment. Is expressed in several structures, including gill; heart; integument; testis; and visual system. |
| *rbm24a* ^11^ | Enables RNA binding activity. Involved in negative regulation of nuclear-transcribed mRNA poly(A) tail shortening and positive regulation of sarcomere organization. Acts upstream of or within several processes, including embryonic organ morphogenesis; epithelial cell differentiation; and mRNA stabilization. Predicted to be located in the cytoplasm. Predicted to be active in cytosol and nucleus. Is expressed in several structures, including cardiovascular system; cephalic musculature; immature eye; segmental plate; and sensory system. |
| *sox2* ^11^ | Enables chromatin binding activity and sequence-specific DNA binding activity. Contributes to DNA-binding transcription factor activity. Acts upstream of or within several processes, including fin regeneration; nervous system development; and otic placode development. Predicted to be located in cytoplasm and nucleus. Predicted to be part of the transcription regulator complex. Is expressed in several structures, including digestive system; immature eye; nervous system; neural keel; and presumptive structure. |
| *Lhx4* ^12^ | Predicted to enable DNA-binding transcription factor activity, RNA polymerase II-specific and RNA polymerase II transcription regulatory region sequence-specific DNA binding activity. Acts upstream of or within ventral spinal cord interneuron specification. Predicted to be active in the nucleus. Is expressed in adenohypophyseal placode; nervous system; spinal cord neural keel; and spinal cord neural tube. |
| *eomesa* ^13^ | Enables DNA binding activity and DNA-binding transcription factor activity, RNA polymerase II-specific. Acts upstream of or within several processes, including determination of dorsal identity; epiboly; and gastrulation. Located in cytoplasm and nucleus. Is expressed in several structures, including blastoderm; blastodisc; brain; eye; and immune system. |
| *dlx5a* ^13^ | Predicted to enable DNA-binding transcription factor activity, RNA polymerase II-specific and RNA polymerase II cis-regulatory region sequence-specific DNA binding activity. Acts upstream of or within several processes, including embryonic morphogenesis; fin development; and olfactory nerve formation. Predicted to be located in the nucleus. Is expressed in several structures, including fin; forebrain; pectoral fin bud; pharyngeal arch; and presumptive structure. |
| *egr1* ^13^ | Predicted to enable several functions, including double-stranded DNA binding activity; promoter-specific chromatin binding activity; and zinc ion binding activity. Acts upstream of or within embryonic retina morphogenesis in camera-type eye; embryonic viscerocranium morphogenesis; and glial cell development. Predicted to be located in cytoplasm and nucleus. Is expressed in head; heart; hindbrain neural keel; mesoderm; and nervous system. |
| *egr4* ^13^ | Predicted to enable DNA-binding transcription factor activity, RNA polymerase II-specific and RNA polymerase II cis-regulatory region sequence-specific DNA binding activity. Predicted to be involved in regulation of transcription by RNA polymerase II. |
| *pde6a* ^14^ | Predicted to enable 3',5'-cyclic-nucleotide phosphodiesterase activity. Predicted to be involved in retina development in camera-type eye and signal transduction. Predicted to act upstream of or within visual perception. Predicted to be active in photoreceptor outer segment membrane. Is expressed in retina; retinal neural layer; retinal photoreceptor layer; and retinal rod cell. |
| *rlbp1a* ^15^ | Predicted to enable phosphatidylinositol bisphosphate binding activity. Acts upstream of or within optokinetic behavior. Is expressed in brain; optic vesicle; and visual system. Human ortholog(s) of this gene implicated in Bothnia retinal dystrophy; Newfoundland cone-rod dystrophy; fundus albipunctatus; night blindness; and retinitis pigmentosa. |
| *rlbp1b* ^15^ | Predicted to enable phosphatidylinositol bisphosphate binding activity. Acts upstream of or within optokinetic behavior. Is expressed in optic vesicle; otic vesicle; pericardial region; pronephric duct; and sensory system. |
| *pdzk1*^16^ | Predicted to enable protein-membrane adaptor activity and signaling receptor binding activity. Predicted to be involved in protein localization to plasma membrane. Predicted to be active in apical plasma membrane. Is expressed in gut; intestinal bulb; pronephric duct; pronephric proximal convoluted tubule; and pronephric proximal straight tubule. |

The descriptions were referred from the Zebrafish Information Network database (https://zfin.org/).

## **Table S4.** Primer pairs used in qRT-PCR analysis.

| **Gnen name** | **Forward primer** | **Reverse primer** |
| --- | --- | --- |
| *β-actin* | CAACAGAGAGAAGATGACACAGATCA | GTCACACCATCACCAGAGTCCATCAC |
| *elavl3* | AGACAAGATCACAGGCCAGAGCTT | TGGTCTGCAGTTTGAGACCGTTGA |
| *adra2b* | CTGGCGCTGGATGTGTTGTTCTG | TTGTTGGCGATCGGGGTGTTT |
| *gap43* | TGCTGCATCAGAAGAACTAA | CCTCCGGTTTGATTCCATC |
| *opn1sw1* | CGATTGCAGGTCTTGTGACG | GACCCTCGGGAATGTATCTGC |
| *rho* | CCCCTCAACTACATCCTGCT | CGACTTTAGCCCCATCTCAC |

## **Table S5.** Thermal procedures of qRT-PCR.

| **Step** | **Temperature** | **Time** |
| --- | --- | --- |
| hold | 95°C | 5 min |
| cycle（40 times） | 95°C | 10 s |
|  | 60°C | 30 s |
| cycle（1 time） | 95°C | 15 s |
|  | 60°C | 60 s |
|  | 95°C | 15 s |

## **Table S6.** The gradient of mobile phase for the instrumental analysis by UPLC-TripleTOF system.

| **Time（min）** | **Mobile phase A**  **0.1% formic acid in water:acetonitrile (95:5, v/v) (%)** | **Mobile phase B**  **0.1% formic acid in acetonitrile:isopropanol:water (47.5:47.5:5, v/v/v) (%)** |
| --- | --- | --- |
| 0 | 100 | 0 |
| 0.5 | 100 | 0 |
| 2.5 | 75 | 25 |
| 9 | 0 | 100 |
| 13 | 0 | 100 |
| 13.1 | 100 | 0 |
| 16 | 100 | 0 |


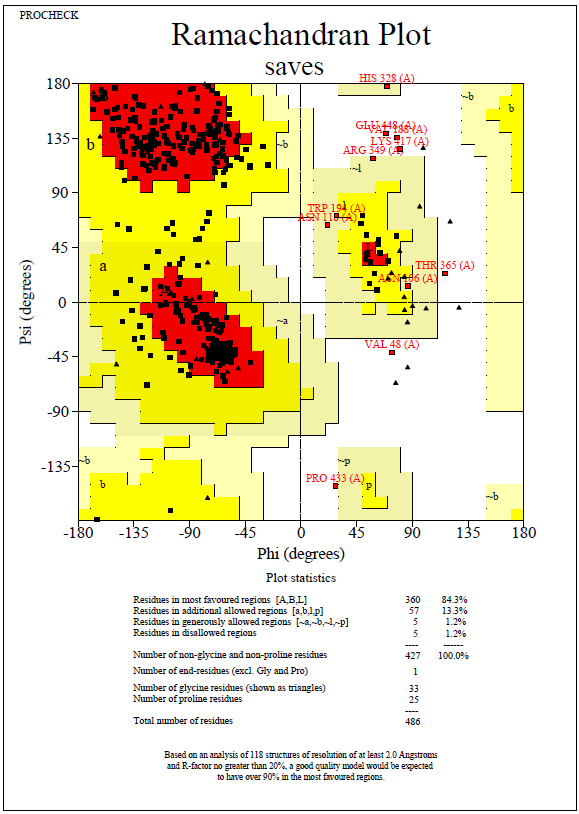


## **Fig. S1.** The modeling quality of nAChR-zebrafish.


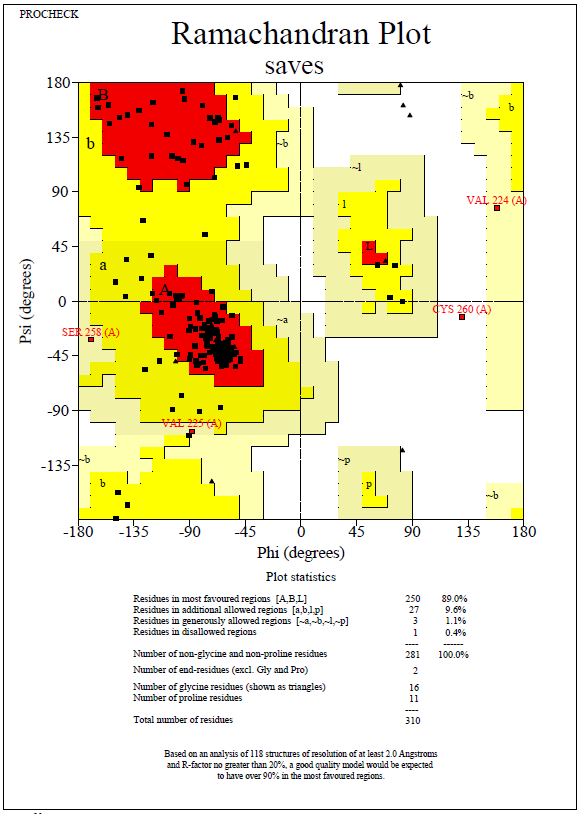


## **Fig. S2.** The modeling quality of GPCR-zebrafish.


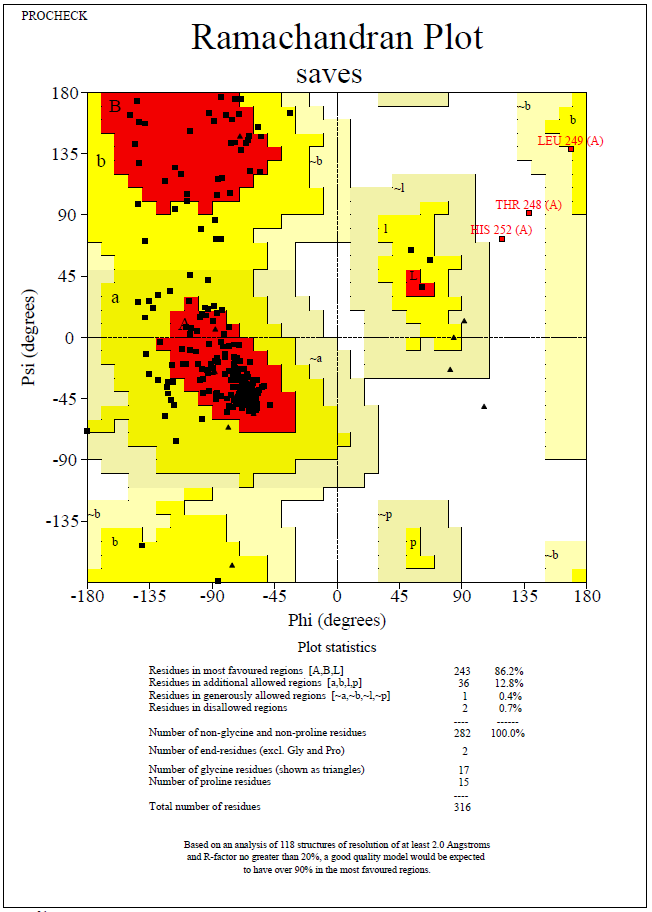


## **Fig. S3.** The modeling quality of ARα-zebrafish.


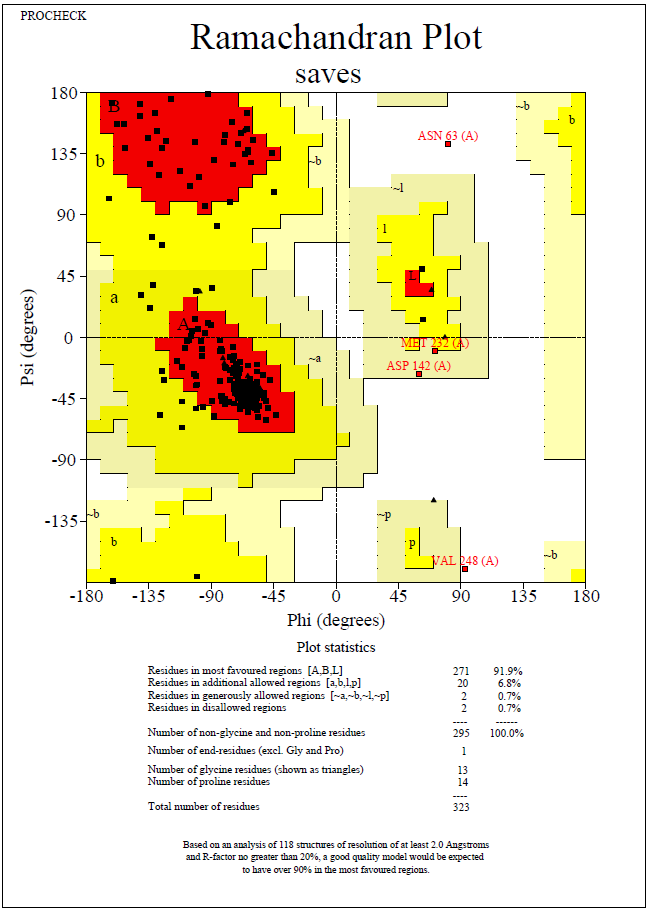


## **Fig. S4.** The modeling quality of ARβ-zebrafish.


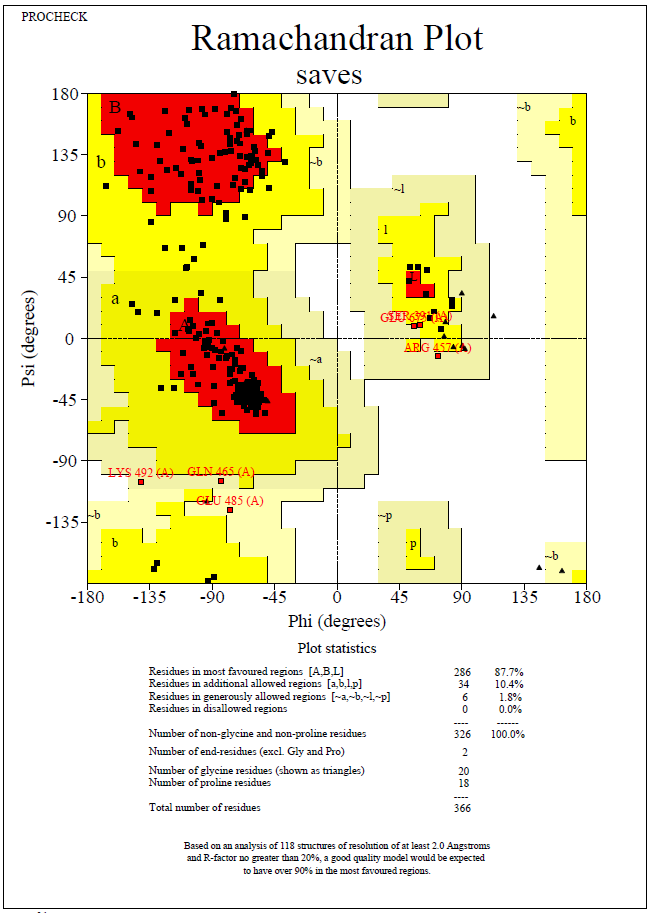


## **Fig. S5.** The modeling quality of GR-zebrafish.


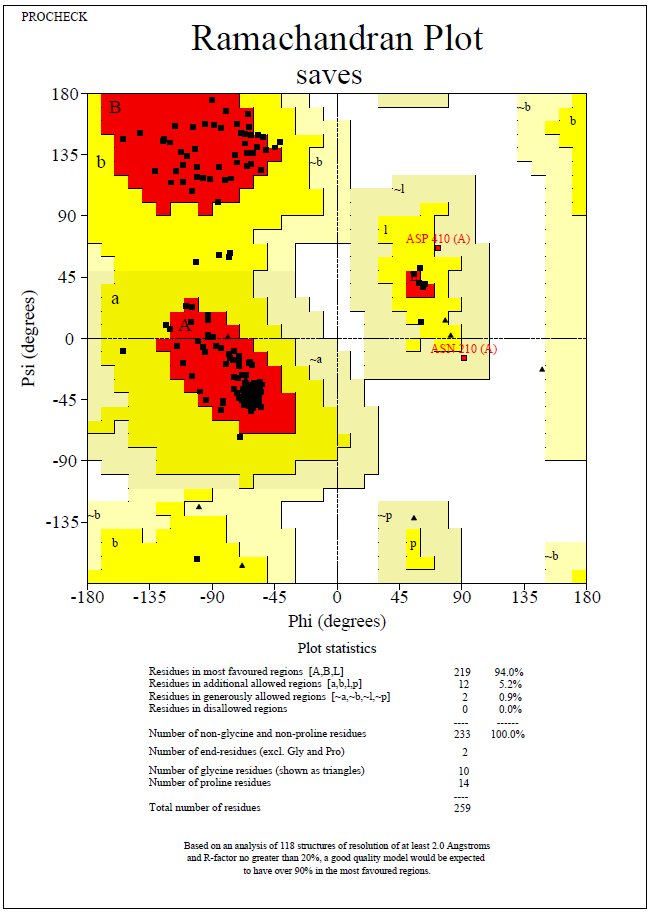


## **Fig. S6.** The modeling quality of TRα-zebrafish.


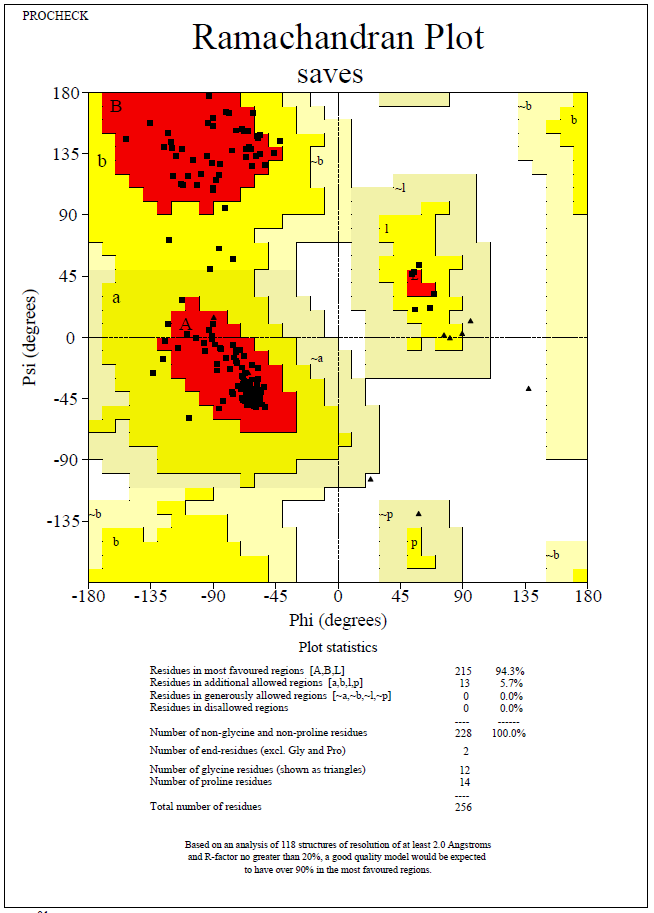


## **Fig. S7.** The modeling quality of TRβ-zebrafish.


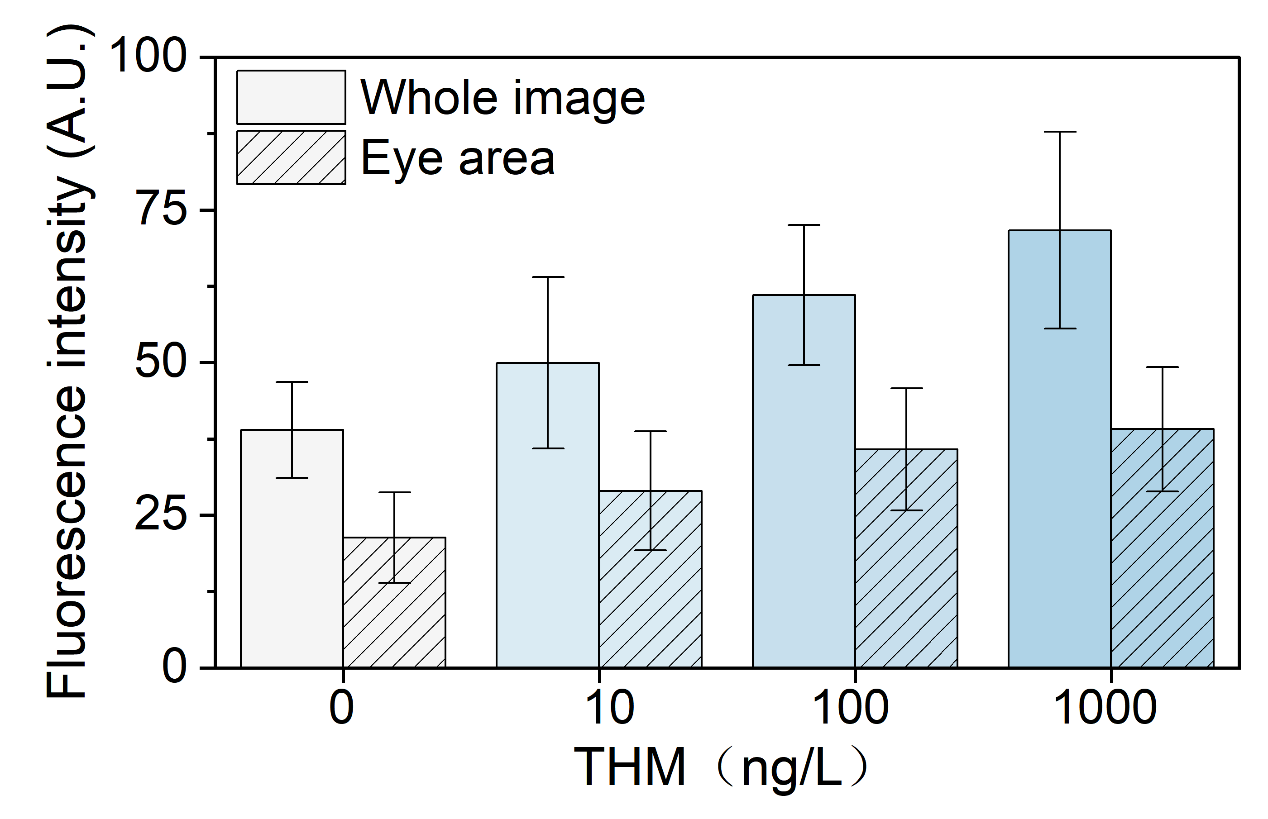


## **Fig. S8.** The fluorescence intensity quantified by Image J.


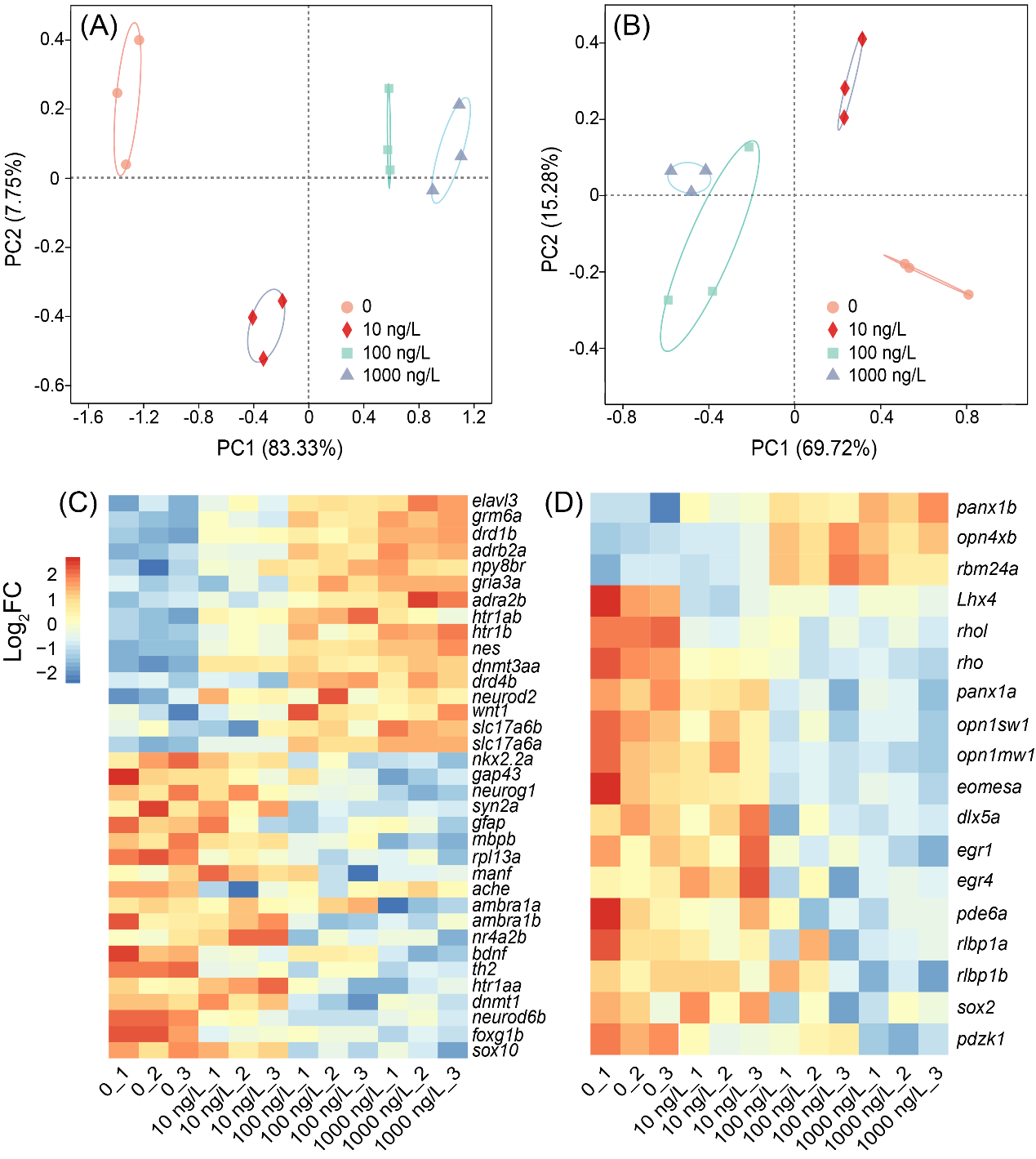


## **Fig. S9.** Principal coordinate analysis (PCoA) of DEGs involve in the neuronal system (A) and visual system(B). Heatmaps of DEGs involved in the neuronal system (C) and visual system (D).


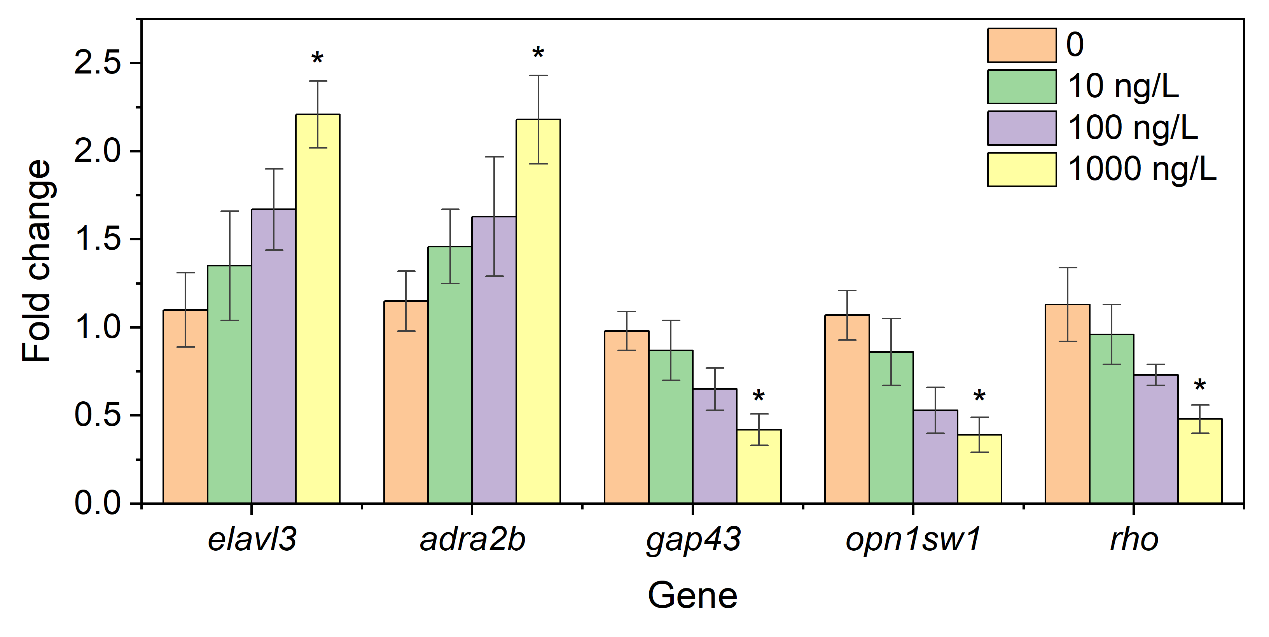


## **Fig. S10.** PCR results of selected genes involved in the neuronal and visual systems. The asterisks (*) represent significant differences between the control group and the experimental groups (*, *p* < 0.05).


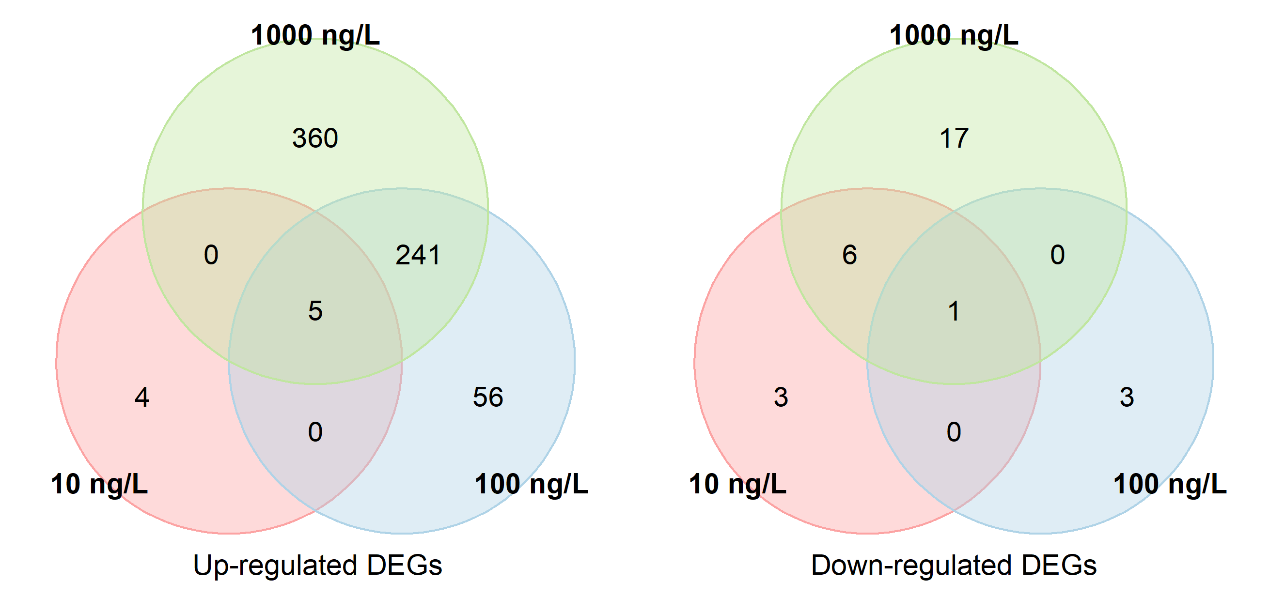


## **Fig. S11.** Venn diagram for significantly up-regulated and down-regulated DEGs after exposure to THM at 10, 100 and 1000 ng/L.


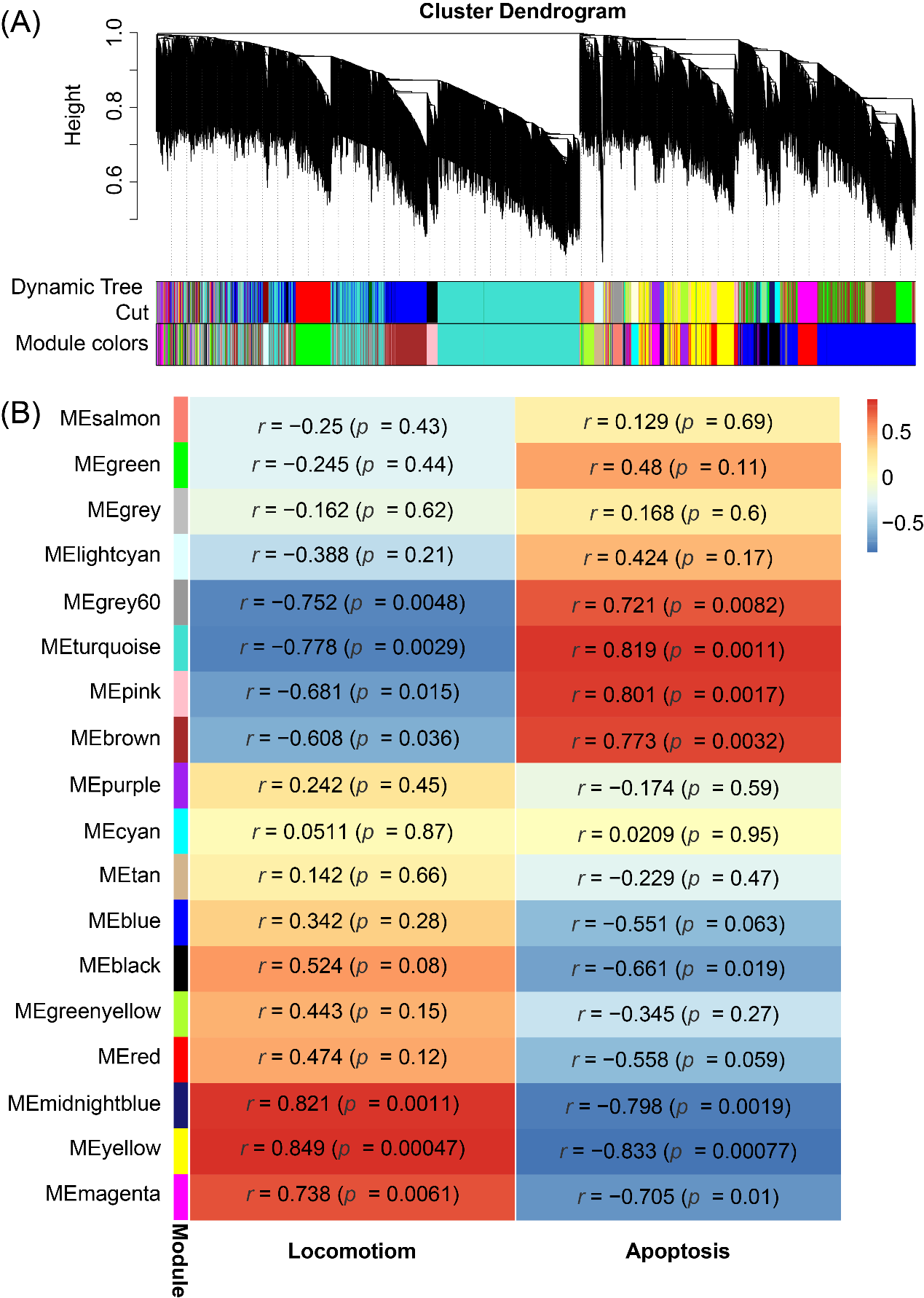


## **Fig. S12.** WGCNA module identification and correlation analysis. The cluster dendrogram of DEGs with dissimilarity based on the topological overlap and assigned module colors (A). Correlation of the identified modules with different traits (B).


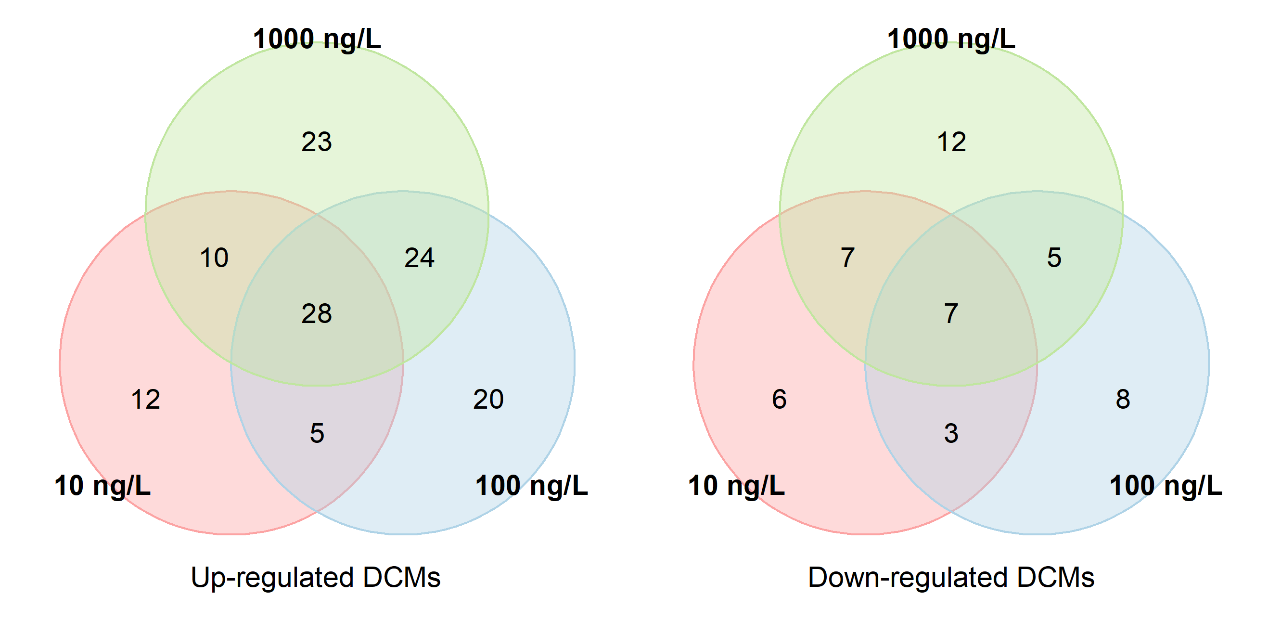


## **Fig. S13.** Venn diagram for significantly up-regulated and down-regulated DCMs.


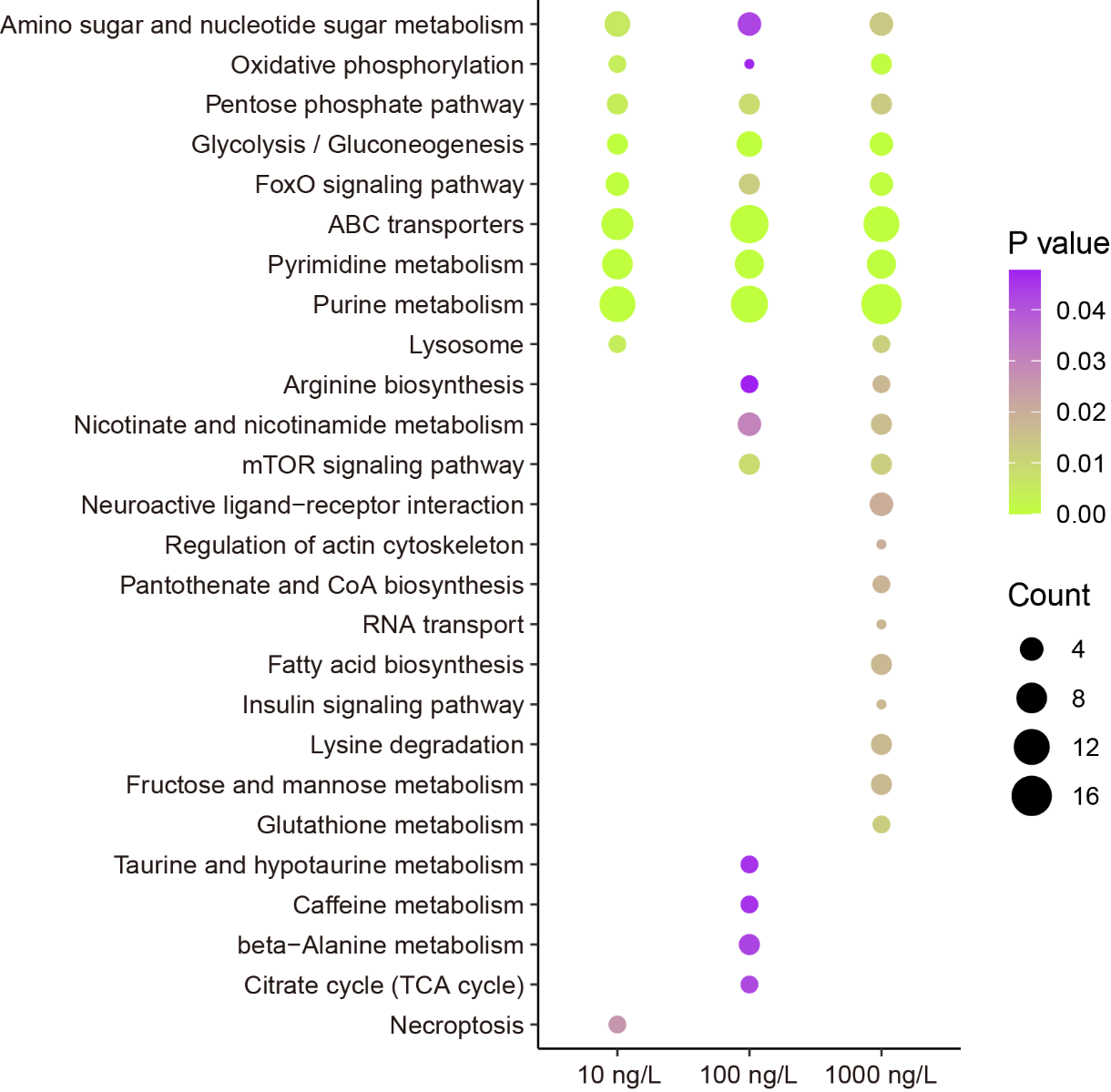


## **Fig. S14.** KEGG pathways of the DCMs after exposure to THM.


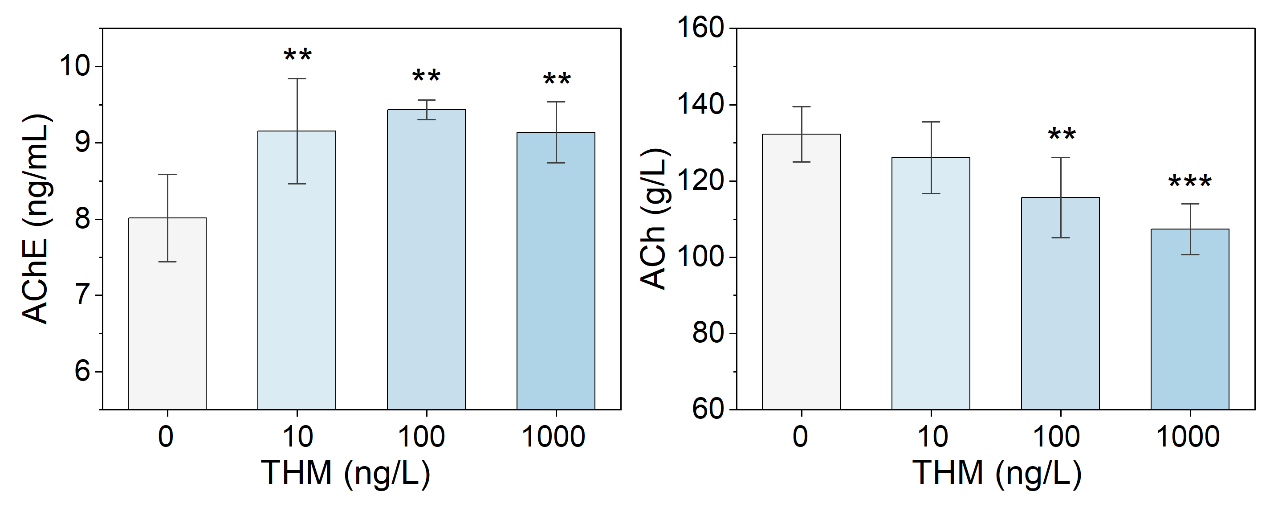


## **Fig. S15.** Alteration on biomarkers in the neuronal system after THM exposure. (A) AChE: acetylcholinesterase, (B) ACh: acetylcholine. The asterisks (*) represent significant differences between the control and the experimental groups (*, *p* < 0.05, **, *p* < 0.01, and ***, *p* < 0.001).


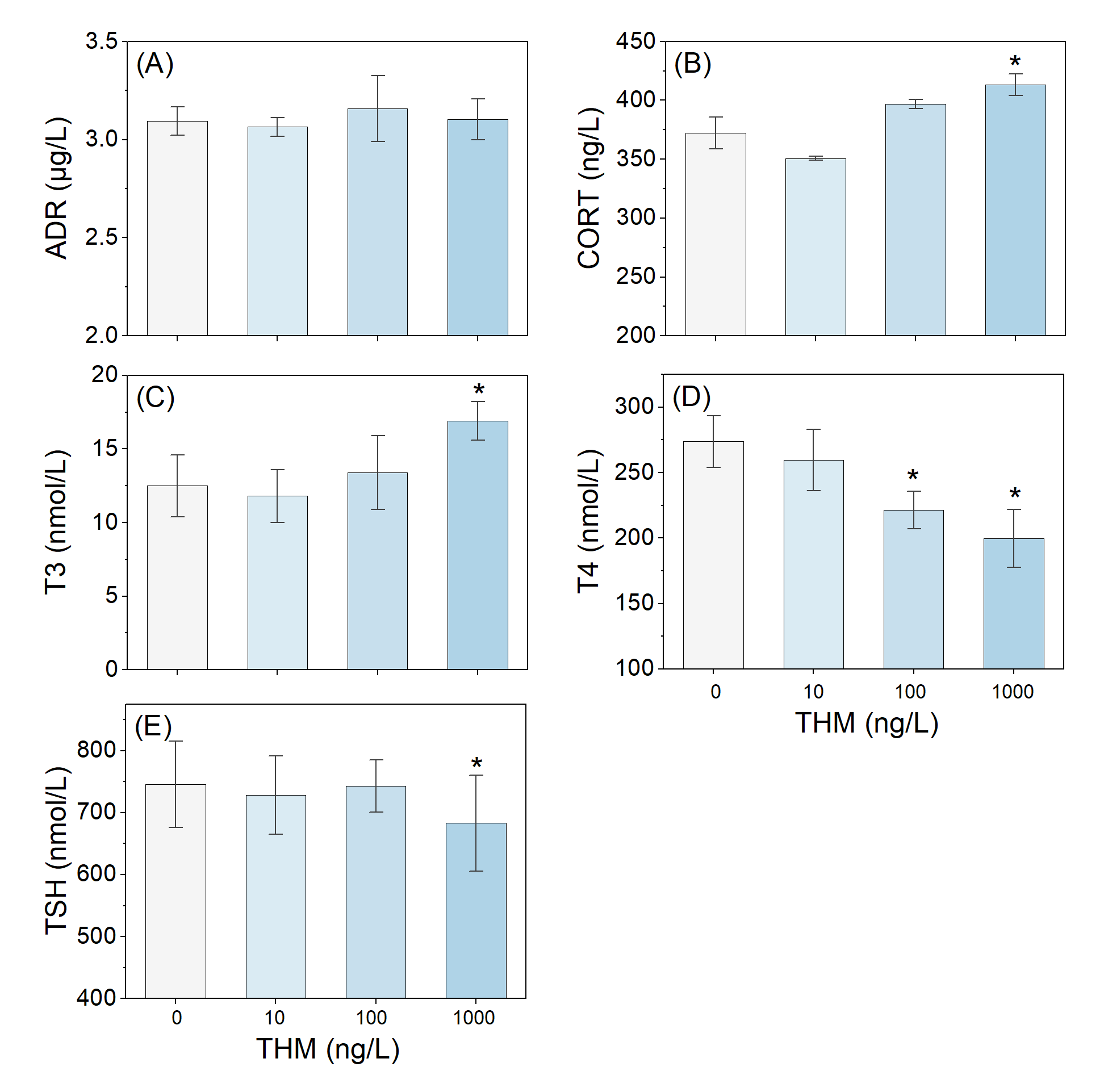


## **Fig. S16.** Alteration on hormones after THM exposure. (A) ADR: adrenaline, (B) CORT: cortisol, (C) T3: triiodothyronine, (D) T4: thyroxine, (E) TSH: thyroid-stimulating hormone. The asterisks (*) represent significant differences between the control and the experimental groups (*, *p* < 0.05, **, *p* < 0.01, and ***, *p* < 0.001).


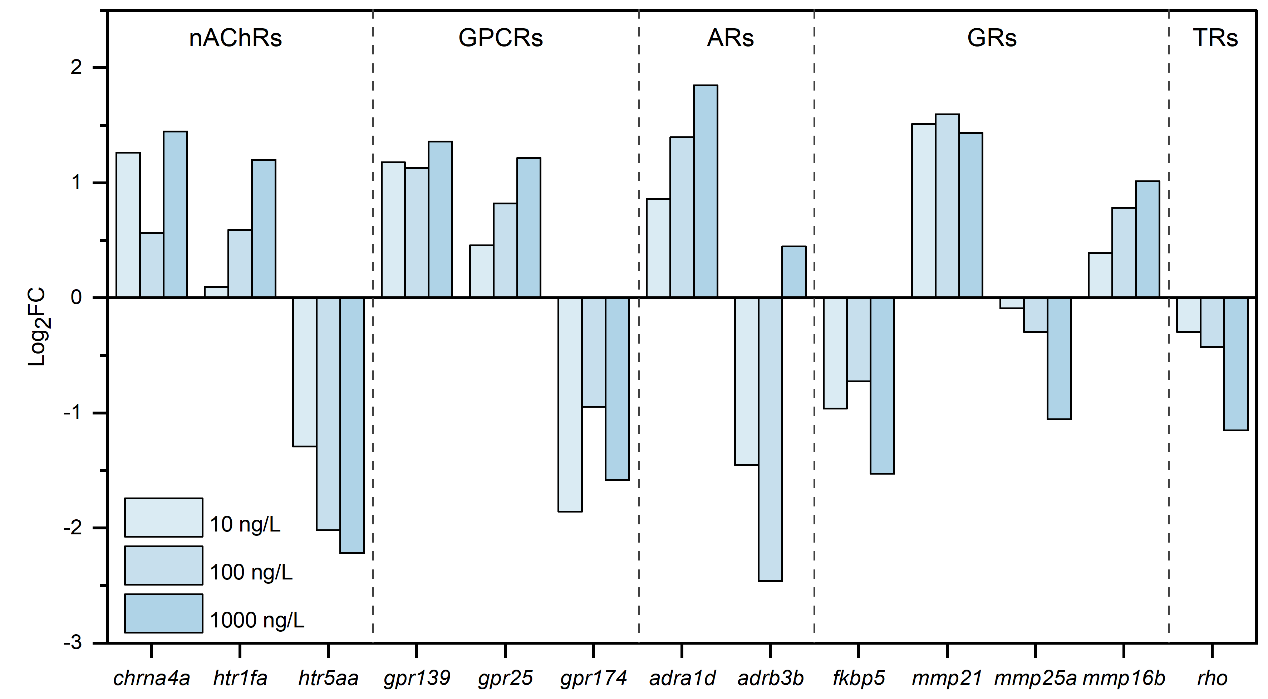


## **Fig. S17.** Expression of genes involved in nAChRs, GPCRs, ARs, GRs, and TRs after THM exposure.


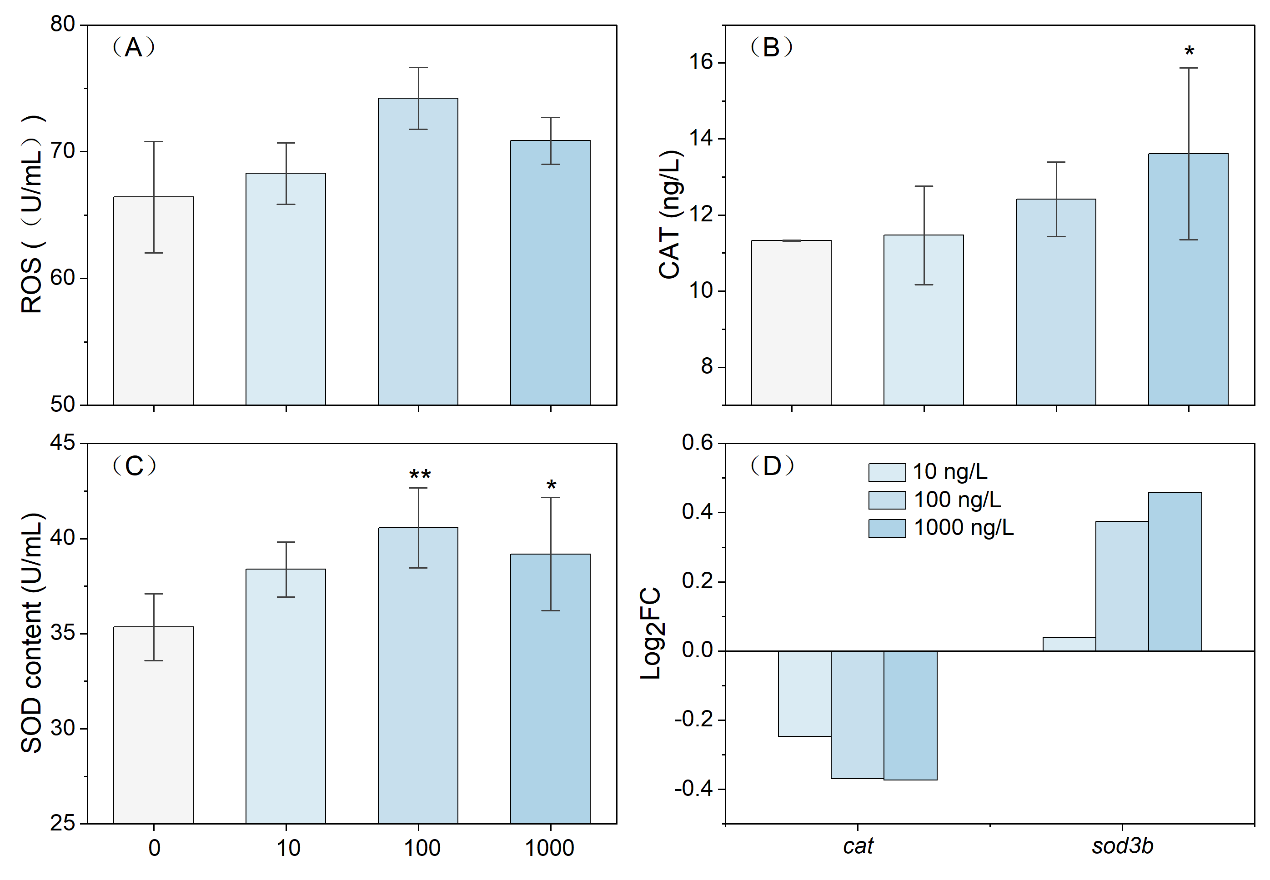


## **Fig. S18.** Alteration on biomarkers and related genes in the antioxidant system after THM exposure. (A) ROS: reactive oxygen species, (B) CAT: catalase, (C) SOD: superoxide dismutase, and (D) Gene expression of *cat* and *sod3b*. The asterisks (*) represent significant differences between the control and the experimental groups (*, *p* < 0.05 and **, *p* < 0.01).

## **REFERENCES**

(1) Li, X.; Zhao, Q.; Li, A.; Jia, S.; Wang, Z.; Zhang, Y.; Wang, W.; Zhou, Q.; Pan, Y.; Shi, P., Spatiotemporal distribution and fates of neonicotinoid insecticides during the urban water cycle in the lower reaches of the Yangtze River, China. *Water Res.* **2022,** *226*, 119232. 10.1016/j.watres.2022.119232

(2) Fan, C. Y.; Cowden, J.; Simmons, S. O.; Padilla, S.; Ramabhadran, R., Gene expression changes in developing zebrafish as potential markers for rapid developmental neurotoxicity screening. *Neurotoxicol. Teratol.* **2010,** *32* (1), 91–98. 10.1016/j.ntt.2009.04.065

(3) Wei, J.; Liu, J.; Liang, S.; Sun, M.; Duan, J., Low-dose exposure of silica nanoparticles induces neurotoxicity via neuroactive ligand-receptor interaction signaling pathway in zebrafish embryos. *Inter. J. Nanomedicine* **2020,** *15*, 4407–4415. 10.2147/ijn.S254480

(4) Jia, M.; Teng, M.; Tian, S.; Yan, J.; Meng, Z.; Yan, S.; Li, R.; Zhou, Z.; Zhu, W., Developmental toxicity and neurotoxicity of penconazole enantiomers exposure on zebrafish (*Danio rerio*). *Environ Pollut* **2020,** *267*. 10.1016/j.envpol.2020.115450

(5) Li, R.; Guo, W.; Lei, L.; Zhang, L.; Liu, Y.; Han, J.; Chen, L.; Zhou, B., Early-life exposure to the organophosphorus flame-retardant tris (1,3-dichloro-2-propyl) phosphate induces delayed neurotoxicity associated with DNA methylation in adult zebrafish. *Environ. Int.* **2020,** *134*. 10.1016/j.envint.2019.105293

(6) Nishimura, Y.; Murakami, S.; Ashikawa, Y.; Sasagawa, S.; Umemoto, N.; Shimada, Y.; Tanaka, T., Zebrafish as a systems toxicology model for developmental neurotoxicity testing. *Congenit. Anom.* **2015,** *55* (1), 1–16. 10.1111/cga.12079

(7) Guo, S. Y.; Zhang, Y.; Zhu, X. Y.; Zhou, J. L.; Li, J.; Li, C. Q.; Wu, L. R., Developmental neurotoxicity and toxic mechanisms induced by olaquindox in zebrafish. *J. Appl. Toxicol.* **2021,** *41* (4), 549–560. 10.1002/jat.4062

(8) Liu, J.; Xu, Y.; Liao, G.; Tu, H.; Huang, Y.; Peng, T.; Chen, X.; Huang, Z.; Zhang, Y.; Meng, X.; Zou, F., The role of ambra1 in Pb-induced developmental neurotoxicity in zebrafish. *Biochem. Biophys. Res. Commun.* **2022,** *594*, 139–145. 10.1016/j.bbrc.2021.12.084

(9) Safarian, N.; Houshangi-Tabrizi, S.; Zoidl, C.; Zoidl, G. R., Panx1b modulates the luminance response and direction of locomotion in the zebrafish. *Inter. J. Mol.r Sci.* **2021,** *22* (21). 10.3390/ijms222111750

(10) Qian, L.; Qi, S.; Wang, Z.; Magnuson, J. T.; Volz, D. C.; Schlenk, D.; Jiang, J.; Wang, C., Environmentally relevant concentrations of boscalid exposure affects the neurobehavioral response of zebrafish by disrupting visual and nervous systems. *J. Hazard. Mater.* **2021,** *404* (Pt A), 124083. 10.1016/j.jhazmat.2020.124083

(11) Brastrom, L. K.; Scott, C. A.; Wang, K.; Slusarski, D. C., Functional role of the RNA-binding protein rbm24a and its target sox2 in microphthalmia. *Biomedicines* **2021,** *9* (2). 10.3390/biomedicines9020100

(12) Guo, R.; Ge, K.; Wang, Y.; Lu, M.; Li, F.; Tian, L.; Gan, L.; Sheng, D., LIM Homeobox 4 (lhx4) regulates retinal neural differentiation and visual function in zebrafish. *Sci. Rep.* **2021,** *11* (1). 10.1038/s41598-021-81211-w

(13) Messina, A.; Potrich, D.; Schiona, I.; Sovrano, V. A.; Fraser, S. E.; Brennan, C. H.; Vallortigara, G., Neurons in the dorso-central division of zebrafish pallium respond to change in visual numerosity. *Cereb. Cortex.* **2022,** *32* (2), 418–428. 10.1093/cercor/bhab218

(14) Crouzier, L.; Diez, C.; Richard, E. M.; Cubedo, N.; Barbereau, C.; Rossel, M.; Delaunay, T.; Maurice, T.; Delprat, B., Loss of pde6a induces rod outer segment shrinkage and visual alterations in pde6aQ70X mutant zebrafish, a relevant model of retinal dystrophy. *Front. Cell Dev. Biol.* **2021,** *9*. 10.3389/fcell.2021.675517

(15) Schlegel, D. K.; Ramkumar, S.; Lintig, J. v.; Neuhauss, S. C., Disturbed retinoid metabolism upon loss of rlbp1a impairs cone function and leads to subretinal lipid deposits and photoreceptor degeneration in the zebrafish retina. *eLife* **2021,** *10*, e71473.

(16) Xie, J.; Jusuf, P. R.; Bui, B. V.; Dudczig, S.; Sztal, T. E.; Goodbourn, P. T., Altered visual function in a larval zebrafish knockout of neurodevelopmental risk gene pdzk1. *Investig. Ophthalmol. Vis. Sci.* **2021,** *62* (3). 10.1167/iovs.62.3.29
